# Unlocking sperm chromatin at fertilization requires a dedicated egg thioredoxin in *Drosophila*

**DOI:** 10.1038/ncomms13539

**Published:** 2016-11-23

**Authors:** Samantha Tirmarche, Shuhei Kimura, Raphaëlle Dubruille, Béatrice Horard, Benjamin Loppin

**Affiliations:** 1 Laboratoire de Biométrie et Biologie Évolutive, Université de Lyon, Université Lyon 1, CNRS, UMR 5558, Villeurbanne F-69622, France

## Abstract

In most animals, the extreme compaction of sperm DNA is achieved after the massive replacement of histones with sperm nuclear basic proteins (SNBPs), such as protamines. In some species, the ultracompact sperm chromatin is stabilized by a network of disulfide bonds connecting cysteine residues present in SNBPs. Studies in mammals have established that the reduction of these disulfide crosslinks at fertilization is required for sperm nuclear decondensation and the formation of the male pronucleus. Here, we show that the *Drosophila* maternal thioredoxin Deadhead (DHD) is specifically required to unlock sperm chromatin at fertilization. In *dhd* mutant eggs, the sperm nucleus fails to decondense and the replacement of SNBPs with maternally-provided histones is severely delayed, thus preventing the participation of paternal chromosomes in embryo development. We demonstrate that DHD localizes to the sperm nucleus to reduce its disulfide targets and is then rapidly degraded after fertilization.

## Abstract (web summary)

In many animals, sperm DNA compaction is achieved by disulfide bonds between the sperm nuclear proteins that replace the histones. Here, the authors provide evidence that *Drosophila* maternal thioredoxin Deadhead is required to unlock the sperm chromatin upon fertilization.

## Graphical abstract

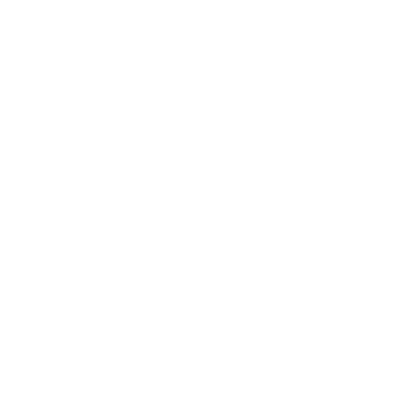

In sexually reproducing animals, the differentiation of haploid spermatids into mature spermatozoa involves major reorganization of the nuclear architecture^1^. Starting as round nuclei after the second male meiotic division, spermatid nuclei slowly transform to eventually acquire the final, species-specific shape of mature sperm nuclei. Extreme compaction of nuclear DNA generally accompanies the streamlining of spermatids, resulting in the shutdown of basic nuclear activities, including transcription and DNA repair^2^. In most species, sperm nuclear compaction requires the massive replacement of somatic-type histones with sperm nuclear basic proteins (SNBPs)^3^,^4^. SNBPs encompass a heterogeneous group of chromosomal proteins that are specifically expressed in male germ cells and deposited during spermiogenesis^3^,^5^. Protamines, the best characterized SNBPs, are small (50–60 aa), arginine rich proteins present in vertebrates and some invertebrates^6^,^7^. A model based on mammalian protamines has proposed that these positively charged proteins bind the major groove of the double helix and form toroid-like structures containing about 50 kilobases of sperm DNA^7^,^8^,^9^. Protamines in eutherian mammals and a few other animal groups are also enriched in cysteine residues, which are otherwise rare in chromosomal proteins, including histones and most SNBPs^3^. During sperm maturation in eutherian mammals, oxidation of the protamine cysteine thiols (–SH) allows the formation of a tridimensional network of disulfide bridges (–S–S–). Intermolecular disulfide crosslinks notably participate in the stabilization of sperm chromatin by connecting adjacent chromatin fibres^7^,^8^,^10^,^11^. It is actually well established that for most species of mammals, a thiol reducing agent, such as dithiothreitol (DTT), is required to elicit sperm nuclear decondensation *in vitro*. In addition, it has been alternatively proposed that in human sperm, protamines thiols are non-covalently bridged by Zinc (Zn^2+^), thereby preventing or limiting the formation of excess disulfide bonds that could perturb sperm nuclear decondensation at fertilization^12^.

In this work, we functionally characterized the *Drosophila* maternal thioredoxin Deadhead (DHD) and we demonstrate that DHD is required to unlock sperm chromatin at fertilization. Thioredoxins are small redox proteins found in all organisms. They typically reduce disulfide bonds on target proteins using a pair of cysteine thiols present in their conserved active CGPC site^13^. Thioredoxins play important metabolic, protective or signalling functions but the molecular bases of their target specificity or functional specialization remain poorly understood^14^. Three classical thioredoxins are found in *Drosophila melanogaster*. Trx-2 is a non-essential ubiquitous protein that participates in the protection against oxidative stress^15^,^16^, whereas TrxT and DHD are sex-specific thioredoxins encoded by a pair of adjacent genes^17^,^18^. While the role of the testis-specific TrxT is unknown, DHD is specifically expressed in the female germline and is essential for embryo development^19^,^20^. In this work, we demonstrate that DHD is essential for sperm nuclear decondensation at fertilization.

## Results

### *deadhead* mutant females produce gynohaploid embryos

The original characterization of the *dhd* maternal effect phenotype showed that the vast majority of eggs produced by homozygous mutant females (hereafter referred to as *dhd* eggs) were fertilized but failed to develop^19^. Interestingly, Salz and colleagues also observed that about 5% of *dhd* embryos reached late embryogenesis and showed severe head developmental defects^19^. We noticed that these defects were reminiscent of the incomplete head involution typically observed in haploid *Drosophila* embryos^21^,^22^,^23^. In addition, early mention of sperm nuclear decondensation defects in *dhd* eggs (see ref. 24) prompted us to reinvestigate the *dhd* phenotype in detail.

**Figure 1:**
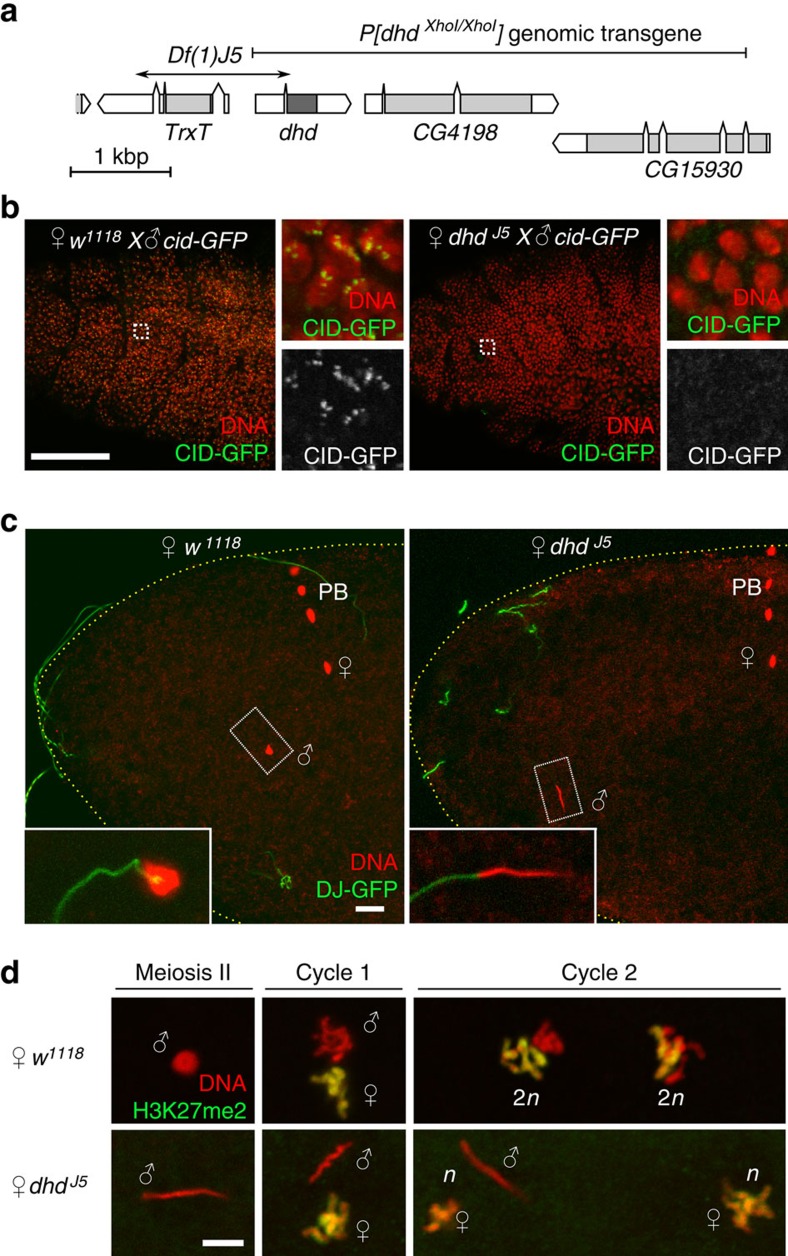
DHD is required for sperm nuclear decondensation. (**a**) The *dhd* genomic region (4F4, X chromosome) showing the genomic *P[dhd*^*XhoI/XhoI*^*]* rescue transgene and the *Df(1)J5* deletion that disrupts *dhd* and *TrxT*. (**b**) Zygotic expression of the paternally-inherited *cid-GFP* transgene, which expresses a GFP-tagged centromeric histone (insets), is detected in late control embryos but not in *dhd* embryos. Scale bar, 50 μm. (**c**) At fertilization, the sperm nucleus (inset) fails to decondense in *dhd* eggs. The sperm flagellum is stained with the Don Juan(DJ)-GFP marker (green). DNA is in red. Note that the four female meiotic products are visible in both eggs. Scale bar, 10 μm. (**d**) The sperm nucleus does not participate in the early zygotic mitoses in *dhd* embryos. Maternal chromosomes are visualized with anti-H3K27me2 staining (green) and appear yellow. PB: polar bodies. Scale bar, 5 μm.

The original *dhd*^*J5*^ allele is a 1.4 kb deficiency on the X chromosome that disrupts *dhd* and the immediately adjacent, paralogous gene *Trx-T*. As *Trx-T* is strictly expressed in the male germline^18^, the *dhd*^*J5*^ allele can be used to specifically address the maternal function of *dhd*^19^. In addition, the *dhd* maternal effect embryonic lethal phenotype is fully rescued by a genomic transgene containing *dhd* but not *Trx-T*^19^ (Fig. 1a; Table 1). To test the possibility that late *dhd* embryos could represent haploid escapers of the early arrest phenotype, we crossed *dhd*^*J5*^ mutant females with males homozygous for a *cid-GFP* transgene. In contrast to embryos from a control cross that zygotically expressed the centromeric marker CID::GFP in all cells, none of the *dhd* embryos that reached gastrulation expressed the paternal marker (Fig. 1b). Direct observations of mitotic figures in *dhd* embryos were also indicative of haploid development (Supplementary Fig. 1). We conclude that rare *dhd* embryos escaping early arrest develop as gynohaploids after the loss of paternal chromosomes.

### *dhd* affects sperm nuclear decondensation

We then examined fertilization and zygote formation in *dhd* eggs to follow the fate of paternal chromosomes. As for other insects, fertilization is internal in *Drosophila* and the needle-shaped, extremely compact sperm nucleus immediately initiates decondensation after its delivery in the egg cytoplasm^25^. Accordingly, during the second female meiotic division (the earliest stage that can be practically observed), the sperm nucleus in wild-type eggs typically appears roundish or at least partially decondensed. In striking contrast, the sperm nucleus systematically retained its original needle-shape in fertilized *dhd* eggs, showing no sign of decondensation (100%, *n*=66; Fig. 1c). Observation of early syncytial divisions in *dhd* embryos confirmed that the paternal genome, still compacted in the sperm nucleus, does not participate in development, thus providing an explanation for the origin of gynohaploid embryos (Fig. 1d).

### The female pronucleus often fails to migrate in *dhd* eggs

**Figure 2:**
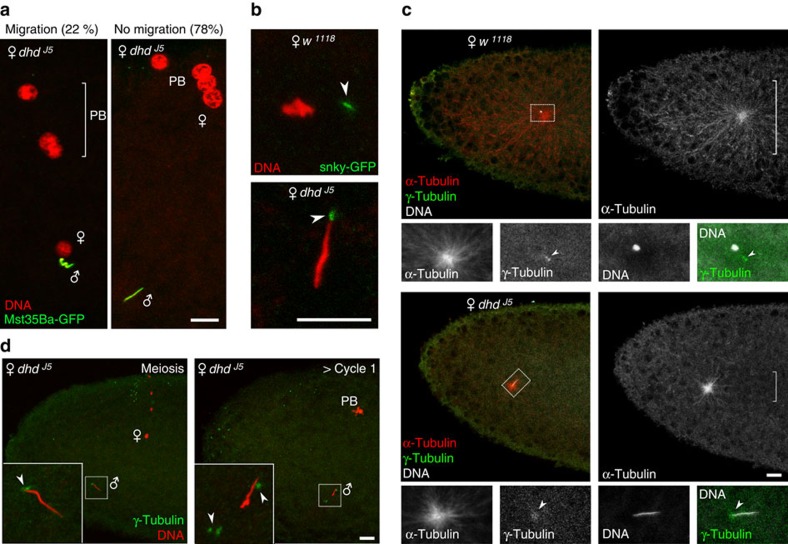
Sperm aster formation and pronuclear migration are affected in *dhd* eggs. (**a**) Left: a *dhd* egg at pronuclear apposition. The three polar bodies (PB) are visible. Right: a *dhd* egg in which the female pronucleus failed to migrate and remained associated with the polar bodies. The respective percentage of each phenotype is indicated (*n*=586). Sperm nuclei were visualized using the paternal *Mst35Ba-GFP* transgene (green). DNA is stained with propidium iodide (red). (**b**) Top panel: a *w*^*1118*^ egg during meiosis II showing the male nucleus and the sperm acrosome (arrowhead). Bottom: a *dhd* eggs during meiosis II with the acrosome still associated to the anterior extremity of the sperm nucleus (arrowhead). The acrosome is visualized with the paternal Snky-GFP marker. (**c**) Eggs in telophase of meiosis II from *w*^*1118*^ and *dhd*^*J5*^ females stained for DNA (white), α-tubulin (red) and γ-tubulin (green) to reveal nuclei, the sperm aster and the centrosomes, respectively (the female meiotic products are not visible on these confocal sections). In *w*^*1118*^ eggs (upper panels), the anti-γ-tubulin stains the zygotic centrosome (which contains a pair of sperm centrioles) at proximity of the male pronucleus (100%, *n*=25; arrowhead, inset). In *dhd* eggs (lower panels), the centrosome appears diffused and almost systematically associated (98%, *n*=53) to the compacted sperm nucleus (arrowhead, inset). The brackets indicate the extent of the sperm aster in *w*^*1118*^ and *dhd*^*J5*^eggs. (**d**) Left: a *dhd* egg in telophase of meiosis II. Right: a *dhd* embryo after the end of cycle 1. The centrosomes (arrowheads, inset) have duplicated and detached from the male nucleus. The rosette of polar body (PB) chromosomes is visible. Green: γ-tubulin, red: DNA. Scale bars, 10 μm.

In *Drosophila*, female meiosis resumes at egg activation, which occurs shortly after ovulation and independently of fertilization. With its two spindles organized in tandem, the second meiotic division generates four meiotic products typically aligned orthogonally to the antero-dorsal egg surface. In fertilized eggs, the innermost meiotic product then migrates towards the male nucleus and becomes the female pronucleus. Pronuclear migration occurs along microtubules of the giant sperm aster, which is nucleated from the paternally-inherited centrioles^25^,^26^. In contrast to the conclusion of the original report^19^, we observed that female meiosis resumed normally (100%, *n*=17) in *dhd* eggs, with the four meiotic products aligned in a way indistinguishable to wild-type eggs (Fig. 1c). We however noticed that, subsequently, the female pronucleus frequently (78%, *n*=586) failed to migrate toward the sperm nucleus and instead remained at the egg periphery with the three other products, as in wild-type unfertilized eggs (Fig. 2a). Pronuclear migration also fails in eggs fertilized by sperm from paternal effect male sterile mutants affecting sperm plasma membrane (SPM) breakdown, such as *sneaky* (*snky*), *misfire* (*mfr*) and *wasted* (*wst*)^27^,^28^,^29^. SPM breakdown is indeed not only critical for sperm nuclear decondensation but is also required for the release of paternal centrioles in the egg cytoplasm and the formation of the sperm aster. To determine if persistence of the plasma membrane on the sperm nucleus could account for the *dhd* phenotype, we crossed control and *dhd* mutant females with males expressing Snky-GFP^30^. Snky-GFP is a specific marker of the sperm acrosome, a membrane-bound vesicular structure at the anterior tip of the sperm nucleus. After fertilization in *Drosophila*, the acrosome normally detaches from the sperm nucleus after the removal of SPM and remains at close proximity in the egg cytoplasm^30^. Interestingly, in *dhd* eggs, the acrosome remained connected to the anterior extremity of the sperm nucleus, opening the possibility that SPM breakdown was not completed in *dhd* eggs (Fig. 2b). Anti-α-tubulin immunostaining however revealed that a sperm aster was systematically present in *dhd* eggs, in sharp contrast to eggs fertilized with *snky* mutant sperm where this structure is never observed (ref. 27 and Supplementary Fig. 2). Although this result demonstrates that SPM breakdown is at least initiated in *dhd* eggs, we noted that the sperm aster appeared abnormally small compared with wild-type eggs observed at the same stage (Fig. 2c). Additionally, we observed that the zygotic centrosome was poorly defined in *dhd* eggs and remained associated to the needle-shape nucleus (Fig. 2d). In early zygotes, however, the centrosome duplicated and eventually detached from the sperm nucleus (Fig. 2d). These observations suggest that the release of paternal centrioles is delayed in *dhd* eggs, which in turn likely affects the recruitment of pericentriolar material and the timely growth of the sperm aster.

### Replacement of SNBPs with histones is delayed in *dhd* eggs

**Figure 3:**
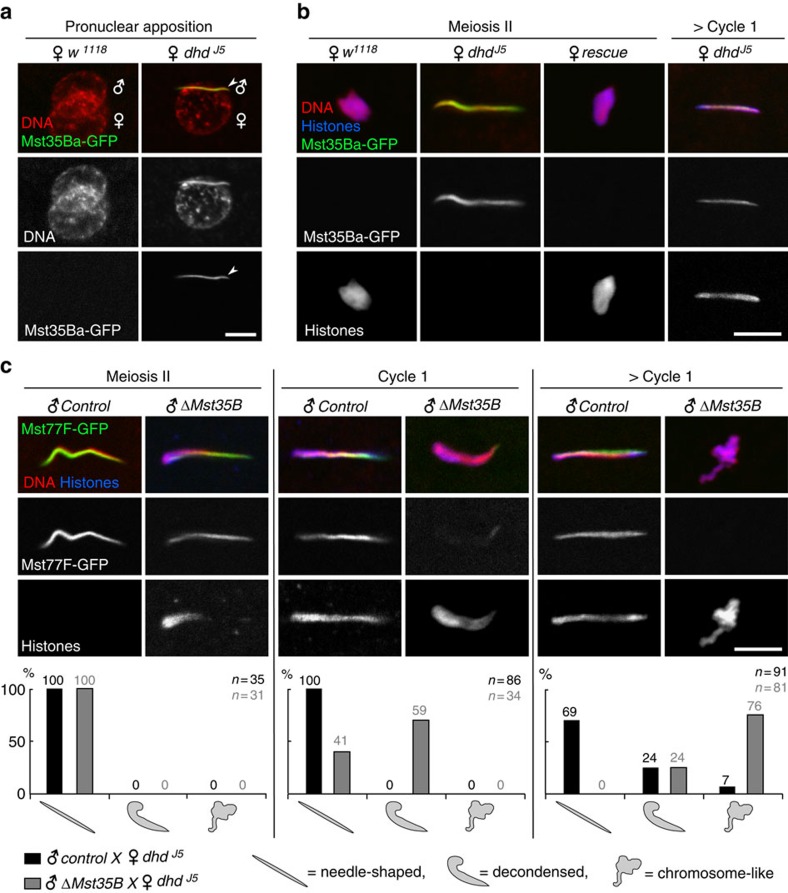
DHD is required for the timely removal of SNBPs at fertilization. (**a**) Pronuclear apposition in eggs from control (*w*^*1118*^) or *dhd*^*J5*^ females mated with transgenic *Mst35Ba-GFP* males. The sperm nucleus (arrowhead) in *dhd* eggs still contains Mst35Ba-GFP (green). (**b**) Eggs or cycle 1 embryos from *w*^*1118*^, *dhd*^*J5*^ or *dhd*^*J5*^*; P[dhd*^*XhoI/XhoI*^*]* (rescue) females mated with *Mst35Ba-GFP* males. The replacement of SNBPs with histones is severely delayed in *dhd* eggs and the phenotype is fully rescued by the genomic transgene. (**c**) Dynamics of SNBP/histone replacement in *dhd* eggs fertilized by control (*Mst77F-GFP*) or mutant (*ΔMst35B ; Mst77F-GFP*) sperm. Distribution of phenotypic classes is shown for each stage. ‘> Cycle 1' indicates embryos with the polar body condensed into a rosette of chromosomes. Scale bars, 5 μm.

*Drosophila melanogaster* sperm nuclei almost entirely lack histones and sperm DNA is instead packaged with at least four SNBPs: the almost identical Mst35Ba and Mst35Bb paralogous proteins (also known as ProtA and ProtB, respectively), Mst77F and the recently identified Prtl99C^31^,^32^. In contrast to protamines, *Drosophila* SNBPs are substantially larger proteins (144–215 aa) that contain equivalent levels of arginine and lysine basic residues. In addition, these protamine-like proteins all possess a motif related to the High Mobility Group (HMG) box found in insects but not in mammals^32^,^33^. At fertilization, *Drosophila* SNBPs are rapidly removed from the sperm nucleus and immediately replaced with maternally-provided histones in a process that requires the nucleosome assembly complex HIRA^34^,^35^,^36^. To analyse the replacement of SNBPs with histones in *dhd* eggs, we crossed mutant females with males expressing GFP-tagged versions of the fly SNBPs (refs 31, 37). Strikingly, we observed that the sperm nucleus systematically retained SNBPs in *dhd* eggs (Fig. 2a) and failed to incorporate histones (100%, *n*=33) (Fig. 3a,b). Importantly, we observed an identical and equally penetrant phenotype in eggs from *dhd*^*J5*^*/Df(1)C70* females, where *Df(1)C70* is a chromosomal deletion that encompasses the *dhd* genomic region (Supplementary Fig. 3; Table 1). Furthermore, this phenotype was fully rescued by a transgene containing the genomic *dhd* sequence (Fig. 3b and Table 1), thus demonstrating that the observed phenotype is caused by the loss of *dhd* function.

Interestingly, in *dhd* embryos that had reached or completed the first zygotic cycle, histones were eventually detected in the sperm nucleus despite the persistence of SNBPs (Fig. 3b and Supplementary Fig. 3). To confirm that this histone staining reflected *de novo* assembly of nucleosomes on paternal chromatin, we performed anti-histone immunostainings on embryos produced by *dhd Hira* double mutant females. We used the *Hira*^*ssm*^ point substitution allele (R225K), which does not affect female viability or fecundity but strongly impacts *de novo* nucleosome assembly in the male pronucleus^34^. In *dhd* embryos observed during nuclear cycle 1 or beyond, histones were almost systematically detected in the needle-shaped sperm nucleus (95.3%, *n*=65). In clear contrast, the sperm nucleus did not contain detectable levels of histones in a majority of *dhd Hira* double mutant embryos observed during the same developmental time window (74.2%, *n*=31), the rest of embryos showing only a very weak staining in the sperm nucleus that we attribute to residual *Hira* activity^34^ (Supplementary Fig. 4). These results confirmed that SNBP/histone replacement is dramatically delayed in *dhd* eggs, thus preventing male pronuclear formation.

To more directly test the possibility that the *dhd* nuclear phenotype was indeed caused by a delay in SNBP removal, we took advantage of the fact that deletion alleles of both *Mst35Ba/b* protamine-like genes do not prevent the production of fertilization-competent sperm^38^,^39^. We reasoned that the previously reported impact of *Mst35Ba/b* deletion on sperm chromatin compaction^32^,^38^ could facilitate the SNBP/histone replacement at fertilization in *dhd* mutant eggs. Crossing *dhd* mutant females with males homozygous for a knock-out of both *Mst35B* copies (*ΔMst35B*) (ref. 39) did not restore embryo viability (Table 1). However, we observed a partial but clear rescue of sperm chromatin remodelling dynamics, as histone deposition was detected before the end of female meiosis in this context (Fig. 3c). In a majority of cycle 1 embryos, sperm nuclei appeared partially decondensed and the SNBP marker Mst77F-GFP was replaced with histones. Finally, in later embryos, the male nucleus frequently (76%, *n*=81) appeared as a mass of chromosome-like structures, a phenotype relatively rare (7%, *n*=91) in control *dhd* embryos collected for the same period of time (Fig. 3c). We conclude that DHD is critically required for the rapid removal of SNBPs from the sperm nucleus at fertilization.

### *Drosophila* sperm chromatin is locked by disulfide bonds

**Figure 4:**
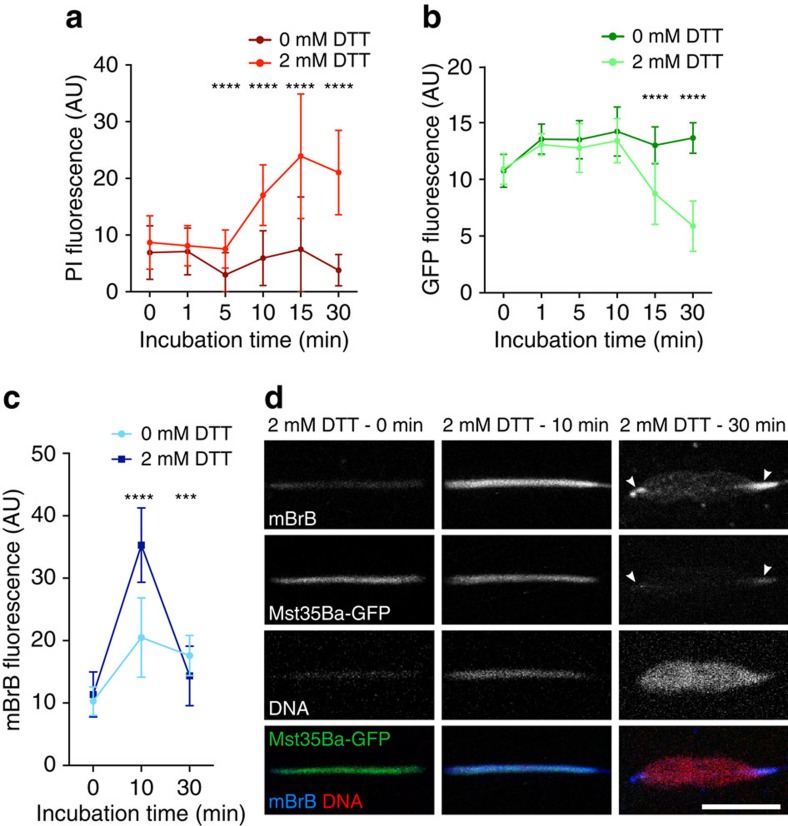
DTT induces SNBP eviction and sperm nuclear decondensation *in vitro*. *Mst35Ba-GFP* sperm dissected from seminal vesicles were incubated with DTT for the indicated time and stained with propidium iodide (PI, red) and mBrB (blue). (**a**–**c**) Quantification of PI (**a**), native GFP (**b**) and mBrB (**c**) fluorescent signals in representative experiments (Mann–Whitney test. ***P<0.001. ****P<0.0001). Error bars indicate SD calculated for 29 analysed nuclei for each time point. (**d**) Confocal images of *Mst35Ba-GFP* sperm nuclei incubated with DTT for the indicated time. Arrowheads indicate condensed nuclear regions positive for both mBrB and Mst35Ba-GFP. Scale bar: 5 μm.

Previous work demonstrated that the typical WCGPCK redox catalytic motif of DHD was essential for its function^20^. We thus wondered whether DHD was involved in the reduction of putative disulfide bonds present on sperm chromatin. Remarkably, all four *Drosophila* SNBPs identified so far show conserved cysteine residues, thus opening the possibility that they could be involved in the formation of disulfide bonds in a way similar to mammalian protamines (refs 31, 32, 40, 41; Supplementary Fig. 5). We thus tested the effect of the disulfide reducing agent DTT on the dynamics of sperm nuclear decondensation *in vitro*. Mature *Mst35Ba-GFP* spermatozoa obtained from dissected seminal vesicles remained basically inert in a non-reducing control buffer, showing no evidence of nuclear decompaction after 30 min of incubation. Strikingly, incubation of sperm nuclei in the presence of 2 mM DTT induced dramatic nuclear decondensation and SNBP removal (Fig. 4). Nuclear swelling appeared maximal in the central region of the nuclei, which also showed the strongest reduction of Mst35Ba-GFP fluorescence. In contrast, both extremities of DTT-treated nuclei remained relatively compacted with a higher density of SNBP (Fig. 4d). Incubation with DTT also increased the amount of free thiol groups in sperm nuclei after 10 min, as measured using the fluorescent marker monobromobimane (mBrB)(Fig. 4c). After 30 min exposure to DTT, however, mBrB fluorescence decreased with the exception of nuclear extremities that still contained higher levels of SNBP. These observations suggest that after the 10 min treatment, mBrB is crosslinked to free thiol groups on SNBPs generated by the reduction of disulfide bonds. A 30 min DTT treatment was however required to induce significant sperm nuclear decompaction and SNBP removal. The eventual decrease of mBrB fluorescence observed after 30 min incubation presumably reflects the eviction of SNBPs. Taken together, our results strongly suggest that *Drosophila* sperm chromatin is stabilized with disulfide bridges, thus opening the possibility that DHD could be required for their reduction at fertilization.

### DHD directly targets sperm chromatin

**Figure 5:**
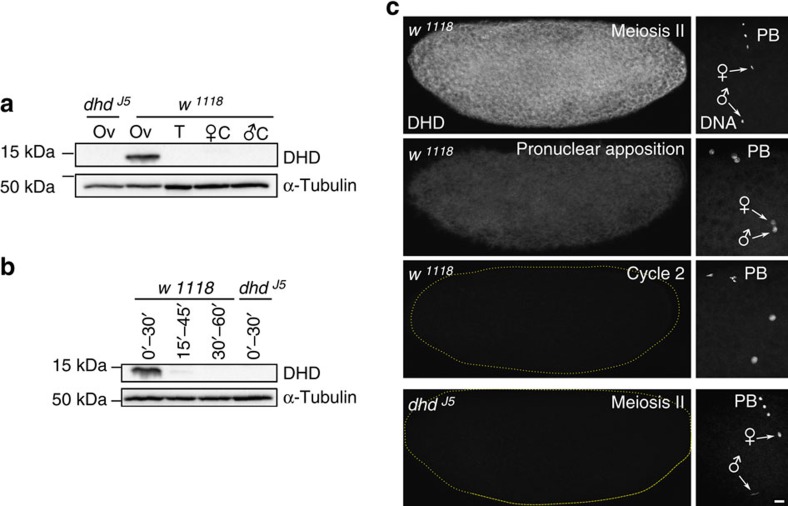
Expression and distribution of maternally-expressed DHD protein. (**a**–**b**) Western blot analyses of DHD expression in adult tissues (a) or embryos collected during the indicated time windows after egg laying (**b**). α-tubulin was used as loading control. Ov: ovaries, T: testis, C: carcasses. See also Supplementary Fig. 6. (**c**) Confocal images of eggs or embryos laid by *w*^*1118*^ or *dhd*^*J5*^ females and stained for DHD (left) and DNA (right). A close-up of the region containing the nuclei is shown on the right (arrows: male and female nuclei; PB: polar bodies). Scale bar, 10 μm

**Figure 6:**
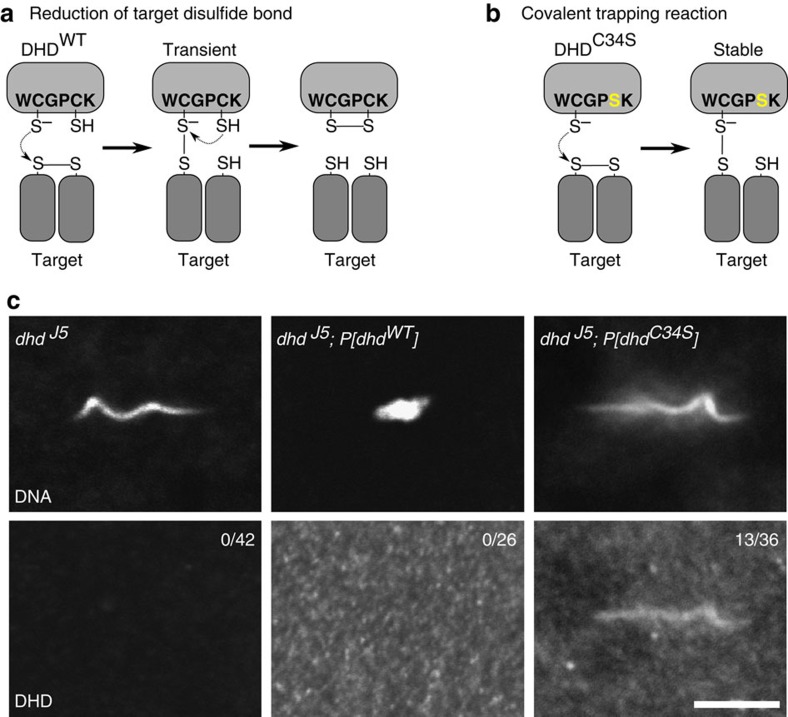
DHD is specifically targeted to the sperm nucleus at fertilization. (**a**) Mechanism of disulfide bond reduction on target proteins by wild-type DHD. (**b**) Strategy for trapping DHD on its targets: the replacement of the resolving cysteine with a serine (C34S) in the WCGPCK motif is predicted to stabilize the mixed-disulfide between DHD and its target. (**c**) Sperm nuclei in eggs laid by females of the indicated genotypes stained for DNA and with the anti-DHD antibody. The number of DHD-positive sperm nuclei is indicated for each genotype. See also Supplementary Fig. 8. Scale bar, 5 μm.

Any direct implication of DHD in the reduction of SNBP disulfide crosslinks would require its preloading in eggs and immediate targeting to the sperm nucleus at fertilization. We first studied the expression and distribution of DHD protein using a specific polyclonal antibody. Western-blotting analyses indeed confirmed that DHD was specifically expressed in ovaries and then maternally deposited in eggs (Fig. 5a,b; Supplementary Fig. 6). At fertilization, DHD appeared very abundant and homogeneously distributed throughout the egg cytoplasm. Surprisingly however, DHD became rapidly undetectable after completion of the first zygotic cycle, thus indicating that this thioredoxin likely plays no other role after zygote formation (Fig. 5c). The rapid removal of SNBPs from the fertilizing sperm nucleus, which is completed before egg deposition^25^, precluded any direct observation of DHD during this process. In an attempt to circumvent this difficulty, we generated transgenic flies expressing a mutated version of DHD (DHD^C34S^), in which the replacement of the second cysteine residue in the WCGPCK catalytic motif is predicted to trap the thioredoxin as a covalent intermediate species with its target proteins^13^ (Fig. 6a,b). In contrast to the fully functional *P*[*dhd*^*WT*^] control transgene, the *P*[*dhd*^*C34S*^] transgene did not rescue the fertility of *dhd* mutant females despite normal expression (Table 1; Supplementary Fig. 7). As expected, in *dhd* eggs rescued with the control transgene, the sperm nucleus appeared normally decondensed and DHD was only detected in the egg cytoplasm. Strikingly however, in about 30% of *dhd*; *P*[*dhd*^*C34S*^] eggs, we observed the specific accumulation of DHD^C34S^ in the needle-shaped sperm nucleus (Fig. 6c; Supplementary Fig. 8). Importantly, DHD^C34S^ was only detected on the sperm nucleus and not on the sperm tail or on the acrosomal region. In the rest of observed eggs, DHD^C34S^ was detected in the cytoplasm but not in the sperm nucleus, again suggesting incomplete sperm plasma membrane breakdown in a fraction of mutant eggs. These results however strongly suggest that DHD directly reduces target disulfides present on sperm chromatin.

### DHD induces sperm nuclear decondensation *in vitro*

**Figure 7:**
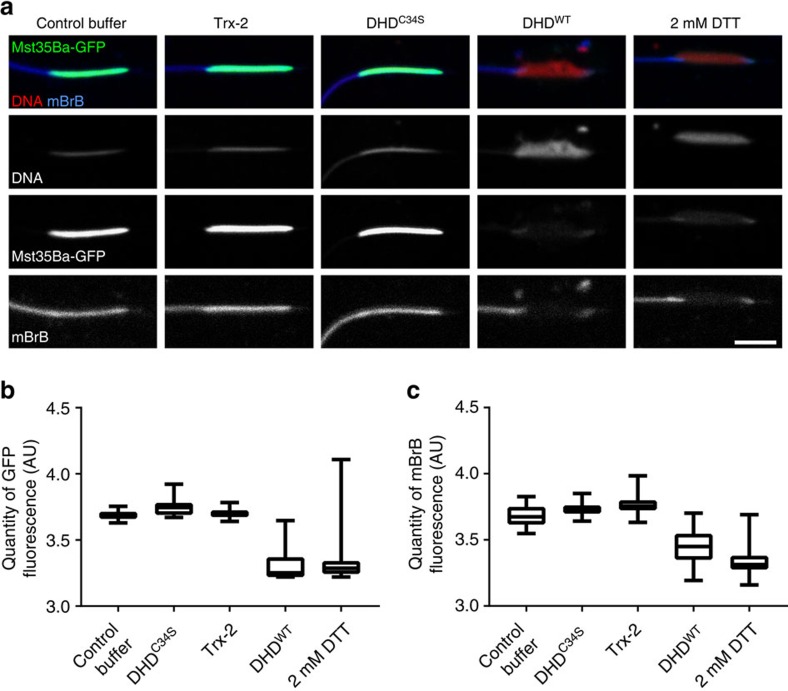
DHD protein is sufficient to decondense sperm nuclei *in vitro.* (**a**) Confocal images of *Mst35Ba-GFP* sperm nuclei incubated with recombinant thioredoxins. The DHD^WT^ recombinant thioredoxin induces nuclear decondensation and Mst35Ba-GFP removal in a way similar to DTT treatment. The sperm nuclei incubated with DHD^C34S^ or Trx-2 recombinant proteins appear similar to sperm nuclei incubated with control buffer. Green: Mst35Ba-GFP, Blue: mBrB, Red: DNA. (**b**,**c**) Quantification of native GFP signal (**b**) or mBrB fluorescence (**c**) from sperm nuclei incubated with the recombinant thioredoxins. Error bars indicate SD calculated for 30 analysed nuclei for each experiment. Scale bar, 5 μm.

To directly address the ability of DHD to perform sperm nuclear decondensation, we tested the activity of purified recombinant thioredoxins on isolated sperm nuclei *in vitro*. We cloned, bacterially-expressed and purified the wild-type and C34S mutant versions of DHD. As an additional control, we also purified Trx-2, the ubiquitously expressed *Drosophila* thioredoxin (Supplementary Fig. 9). The 6-histidine-tagged purified thioredoxins were reduced using DTT before their use in the assay. Incubation of Mst35Ba-GFP sperm nuclei with either Trx-2 or DHD^C34S^ had no detectable effect on sperm nuclear compaction or the level of Mst35Ba-GFP fluorescence. In clear contrast, incubation of sperm nuclei with wild-type recombinant DHD protein induced both sperm nuclear decondensation and Mst35Ba-GFP removal in a way similar to the effect of 2 mM DTT treatment (Fig. 7). These experiments strongly support a direct role of DHD in SNBP removal and sperm nuclear decondensation. Furthermore, the lack of detectable activity of Trx-2 in this assay suggests that DHD presents unique properties not shared with other thioredoxins.

## Discussion

In this study, we have identified the *Drosophila* maternal thioredoxin Deadhead as critically required for sperm nuclear decondensation at fertilization. We propose that DHD is involved in the reduction of disulfide crosslinks that connect cysteine residues of SNBPs in mature sperm chromatin.

The stabilization of sperm chromatin with disulfide bonds is a remarkable innovation that emerged independently in distantly-related animal groups with the selective acquisition of cysteine residues in SNBPs^3^,^42^,^43^. In this work, we first established that *Drosophila* sperm chromatin, which is packaged with cysteine-rich SNBPs, is stabilized by disulfide crosslinks. We have shown that the reducing agent DTT allows efficient decompaction of *Drosophila* sperm nuclei *in vitro*, in a way similar to its well-established activity on sperm nuclei from eutherian mammals, for instance^44^. Similarly, it has been recently shown that pre-treatment of *Drosophila* sperm nuclei with DTT improves antibody accessibility to nuclear epitopes in immunofluorescence experiments^32^. Although little is known about the structural organization of *Drosophila* sperm chromatin, a recent study showed that the *Drosophila* SNBP Mst77F is a DNA binding protein which induces DNA condensation *in vitro* through a multimerization process involving its coiled-coil domain^45^. Oxidation of cysteine thiols present in Mst77F could participate in the stabilization of this unique chromatin architecture through the establishment of disulfide crosslinks. Disulfide bonds could also allow the formation of inter-chromatin fibre associations, as proposed for mammalian sperm chromatin^8^.

The implication of DHD in the reduction of sperm chromatin disulfide crosslinks in *Drosophila* eggs is first supported by our detailed characterization of the *dhd* phenotype. Among the rare *Drosophila* maternal effect mutants affecting sperm chromatin remodelling^33^,^34^,^46^, *dhd* is unique and shows the earliest phenotype reported so far. Notably, in *dhd* mutant eggs, the sperm nucleus is initially intact, showing no sign of decondensation. Second, any role of DHD in the reduction of sperm nuclear disulfide bonds implies the critical requirement of its catalytic redox motif. Salz and colleagues previously demonstrated that transgenes expressing DHD in which the two active cysteines are replaced by serines failed to rescue the fertility of mutant females^20^. In this work, we have shown that replacing the second cysteine residue of the redox motif not only abolished DHD function but also trapped the protein on sperm chromatin in a remarkably specific manner. This experiment strongly suggests that DHD directly targets disulfide bonds on sperm chromatin. Interestingly, the DHD^C34S^ mutant protein could not rescue the *dhd* nuclear phenotype despite its expected ability to cleave its disulfide targets (Fig. 6b). We speculate that the irreversible covalent trapping of DHD^C34S^ on SNBPs at the surface of sperm chromatin could impede their eviction and prevent further access of the thioredoxin to the compact sperm nucleus. We thus propose that, following sperm penetration through the egg micropyle and sperm plasma membrane breakdown, the sperm nucleus is exposed to the egg cytoplasm that contains massive amounts of DHD thioredoxin. The reduction of disulfide crosslinks by DHD induces sperm nuclear decompaction and allows the subsequent extraction of SNBPs from sperm chromatin, a process that likely involves the ISWI chromatin remodeler^33^. Finally, the removal of SNBP leaves DNA accessible to the HIRA complex for genome-wide nucleosome assembly with maternally supplied histones^34^,^35^,^36^.

In mammals, the tripeptide antioxidant glutathione is abundant in oocytes and plays an important role in sperm nuclear decondensation^44^,^47^,^48^. Inhibition of glutathione synthesis by buthionine sulfoximine during oocyte maturation indeed efficiently blocks sperm nuclear decondensation and this effect can be reversed by DTT (refs 47, 48). Whether glutathione directly reduces SNBP disulfides *in vivo* or instead activates a dedicated disulfide reductase is still unresolved^49^. In most species, glutathione disulfide (GSSG) is reduced into glutathione (GSH) by the highly conserved glutathione reductase enzyme. *Drosophila* is unusual in lacking glutathione reductase and instead relies on the thioredoxin system for GSSG reduction^50^. Interestingly, biochemical characterization of *Drosophila* thioredoxins have established that DHD and Trx-2 are equally efficient for GSSG reduction *in vitro*^50^,^51^, opening the possibility that DHD could indirectly influence sperm nuclear decondensation by controlling the level of GSH in eggs. However, our demonstration that recombinant DHD protein is sufficient for sperm nuclear decondensation and SNBP removal in our *in vitro* assay argues against this hypothesis. Still, we have clearly documented that sperm chromatin remodelling is not completely blocked in *dhd* eggs but instead occurs very slowly. This slow replacement of SNBPs with histones suggests that mutant eggs still have residual reducing power for the cleavage of SNBP disulfide bonds, an effect that could likely involve glutathione itself.

Our study also revealed that the migration of the female pronucleus to the centre of the egg fails in a majority of *dhd* eggs, thus preventing the initiation of embryo development. We found that the release of paternal centrioles from the base of the sperm nucleus and the subsequent elaboration of the sperm aster were indeed affected in mutant eggs. As a matter of fact, depletion of glutathione in bovine oocytes not only blocks sperm nuclear decondensation but also prevents sperm aster growth and pronuclear migration^48^. The authors indeed observed that the disassembly of the sperm tail and connecting piece, as well as the release of the proximal centriole were frequently affected in treated oocytes, suggesting that the role of glutathione at fertilization is not restricted to sperm chromatin decompaction. Further investigation will be required to determine whether DHD similarly targets additional sperm structures.

In conclusion, we propose that the rapid unlocking of sperm chromatin at fertilization in *Drosophila* is critically dependent on the redox activity of the egg specific DHD thioredoxin. *dhd* is apparently the most recent thioredoxin gene in *Drosophila* and likely originated after the duplication of the ancestral *Trx-2* gene^18^. It is thus tempting to propose that the emergence of this highly specialized protein could reflect the adaptation of the oocyte to the rapid evolution of sperm chromatin architecture. Future work will aim at understanding how the structural features of DHD underlie its crucial specialization at the onset of embryo development.

## Methods

### Fly stocks

Flies were raised at 25 °C on standard cornmeal-agar-yeast medium. The *w*^*1118*^ strain was used as a wild-type control. The *Df(1)J5/FM7c* and *Df(1)J5/Df(1)J5; CyO, P*[*dhd*^*XhoI/XhoI*^*, w*^*+*^]*/+* stocks^19^ were obtained from H. Salz. The boundaries of the genomic fragment carried by *P[dhd*^*XhoI/XhoI*^*, w*^*+*^] transgene (see Fig. 1a) were verified by PCR and DNA sequencing. We additionally used the following stocks: *P[snky-GFP]; snky*^*1*^ (ref. 30), *P[EGFP-cid]* (ref. 52), Δ*Mst35B* (ref. 39), *P*[*Mst35Ba-EGFP*] (ref. 37), *P*[*Mst77F-EGFP*] (ref. 31), *snky*^*1*^ (ref. 27) and *P*[*Dj-GFP*] (ref. 53). Additional stocks were obtained from the Bloomington *Drosophila* stock centre: *Df(1)JC70* (#944) is a deficiency of the cytogenetic region 4C11-5A4 which does not complement the female sterility associated with *dhd*^*J5*^ (Table 1); *Df(3L)BSC12* (#6457) is a deficiency that uncovers the *snky* locus. The *pW8-attB[dhd*^*WT*^] and *pW8-attB*[*dhd*^*C34S*^] transgenic lines were generated by PhiC31 mediated insertion^54^ into the *PBac{y*^*+*^*-attP-3B}VK00031* platform (62E1).

### Fertility tests

Virgin females were aged for 3 days at 25 °C in the presence of males and were then allowed to lay eggs on standard medium for 24 h. Embryos were counted and then let to develop at least 36 h at 25 °C. Unhatched embryos were counted to determine hatching rates.

### Plasmid constructs

#### pW8-attB[dhd^WT^]

A 4303 bp genomic fragment (*dhd*^*WT*^) containing the *dhd* coding sequence, upstream (1932 bp) and downstream (2047 bp) regions, was amplified from wild-type genomic DNA using the following primers: 5′-GCCGGTACCCCCATATCCCTCCCATATCC-3′ and 5′-GCCGGATCCGCTAATGGAATCGCAATCGT-3′ and cloned into the *pW8-attB* plasmid^55^.

#### pW8-attB[dhd^C34S^]

The *dhd*^*C34S*^ fragment was obtained by *in vitro* mutagenesis of *dhd*^*WT*^ plasmid using the QuickChange II XL Site-Directed Mutagenesis Kit (Agilent Technologies) with the following primers: 5′-CATGGTGTGGTCCCAGCAAGGAAATGGAGAGC-3′ and 5′-GCTCTCCATTTCCTTGCTGGGACCACACCATG-3′ and cloned into *pW8-attB*.

#### pET15B(dhd^WT^) and pET15b(dhd^C34S^)

The *dhd*^*WT*^ and *dhd*^*C34S*^ fragments were amplified from the *pW8-attB(dhd*^*WT*^) and *pW8-attB(dhd*^*C34S*^) plasmids, respectively, with the following primers 5′-TAATTCCATATGATGGCATCCGTACGCACCATG-3′ and

5′-TAATTCGGATCCTTACGCCTTCACCAGCTTGGC-3′ and cloned into *pET15b*.

#### pET15b(Trx-2)

The *Trx-2* coding sequence was synthetized, amplified with the following primers 5′-TAATTCCATATGATGGTGTACCAGGTGAAAGAT-3′ and 5′-TAATTCGGATCCTTAGATATTGGCCTTGATGACA-3′ and cloned into *pET15b*.

### DHD antibody production

A rabbit polyclonal anti-DHD antiserum was raised against a mixture of three synthetic peptides (DHD-1: MASVRTMNDYHKRIEAADC, DHD-2: CKEMESTVKSLARKYSSKA, DHD-3: CAGADEHKLTNMMAKLVKA). DHD antibody was purified by affinity chromatography.

### Embryos preparation and imaging

Early (0–30 min) and late (0–120 min) embryos were collected on agar plates. Embryos were dechorionated in bleach, fixed in a 1:1 heptane:methanol mixture and stored at −20 °C. For immunostaining experiments, embryos were washed three times (10 min each) in PBS, 0.1% Triton X100. Embryos were incubated with primary antibodies in the same buffer on a wheel overnight at 4 °C and washed three times (20 min each) in PBS, 0.1% Triton X100. Incubation with secondary antibodies was performed identically. For propidium iodide staining, embryos were incubated for 1 h in a 2 mg per ml RNAse A solution at 37 °C. Embryos were finally mounted in mounting medium (Dako) containing propidium iodide (Sigma) or DAPI.

Primary antibodies used were: mouse monoclonal anti-GFP (Roche #118144600001; 1:200), rabbit polyclonal anti-H3K27me2 (Millipore #07-452; 1:500), mouse monoclonal anti-histones (Millipore #MABE71; 1:1,000), mouse monoclonal anti-γ-Tubulin (Sigma #T6557; 1:500), rat monoclonal anti-α-Tubulin (Abcam #ab6160; 1:50), rabbit polyclonal anti-DHD (1:1,000). Secondary antibodies were used at a 1:1,000 dilution and included goat anti-mouse and goat anti-rabbit antibodies conjugated to DyLight (Thermoscientific), as well as goat anti-rat and goat anti-mouse conjugated to AlexaFluor (Jackson ImmunoResearch). Images were acquired on a LSM 510 META (Carl Zeiss) or a TCS SP5X (Leica) confocal microscope and the images were processed with ImageJ and Inkscape softwares.

### Expression and preparation of recombinant thioredoxins

*E. coli* BL21 cells (Agilent Technoligies #230134) were transformed with the different pET15b constructs and their expression were induced using 1 mM IPTG (isopropyl β-D-thiogalactoside) for 2 h at 37 °C. Cell lysis and protein purification were performed with the Protino Ni-NTA agarose kit (Macherey-Nagel) according to the manufacturer instructions. Before incubation with sperm nuclei, the recombinant thioredoxins were reduced for 3 h at room temperature with 10 mM DTT and desalted with PD-10 Desalting Column containing Sephadex G25 Medium (GE Healthcare) to remove excess DTT. Protein elution was performed using 0.1 M Potassium Phosphate Buffer pH 7.0, 1 mM EDTA. The control buffer used in decondensation assays is 0.1 M Potassium Phosphate Buffer pH 7.0, 1 mM EDTA; 10 mM DTT eluted from PD10 Desalting column.

### Preparation and imaging of sperm nuclei treated with DTT

Virgin males were collected five days before dissection. Seminal vesicles were dissected in 1X PBS; 1% Triton X100 and torn open with tweezers to release spermatozoa on polylysine slides. Slides were first incubated at room temperature in 1X PBS; 1% Triton X100 for 30 min, air dried and then incubated in 1X PBS; 2 mM DTT for 0, 1, 5, 10, 15 or 30 min at room temperature. Slides were quickly washed in 1X PBS, incubated for 15 min at room temperature in 1mM mBrB (Millipore #596105), quickly washed and mounted in mounting medium (Dako) containing propidium iodide (Sigma).

### Decondensation of sperm nuclei with recombinant thioredoxins

Seminal vesicles from virgin males were dissected as described above. Slides were incubated at room temperature in 1X PBS 1%, Triton X100 for 30 min, air dried and then incubated 3 times for 30 min in Potassium Phosphate Buffer containing the desalted thioredoxins. Slides were quickly washed in 1X PBS. Before quantification, slides were incubated for 15 min at room temperature in 1 mM mBrB (Millipore #596105), quickly washed and mounted in mounting medium (Dako) containing propidium iodide (Sigma).

### Imaging and fluorescence quantification of sperm nuclei

Sperm nuclei were imaged on a TCS SP5 X confocal microscope (Leica) or on a LSM 800 confocal microscope (Carl Zeiss). The images were analysed using the ImageJ 1.48 software to measure the total quantity of fluorescence. Each nucleus was counted as an individual object in a defined area and the background signal outside the nucleus was used to determine the fluorescence baseline. Within the selected area, the number of pixels for each value of fluorescence (ranging from the baseline to 255) was counted. Finally, the total quantity of fluorescence was calculated with the following formula:


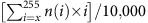
 in which *i* is the fluorescence value, *x* is the baseline fluorescence and *n(i)* is the number of pixels for the fluorescence value *i*.

PI and GFP signals were quantified in the same experiment and mBrB signal in an independent one. Each experiment was performed twice and gave similar results. Statistical tests (Mann Whitney) were performed using Prism (Graphpad) software.

### Western Blotting

Ovaries or testes from 30 individuals were collected and homogenized in lysis buffer (20 mM Hepes pH 7.9, 100 mM KCl, 0.1 mM EDTA, 0.1 mM EGTA, 5% Glycerol, 0.05% NP40 and protease inhibitors (Roche)). The protein extracts were cleared by centrifugation and stored at −80 °C.

Eggs were collected every 30 min and incubated for 0, 15 or 30 min at 25 °C. They were then dechorionated in bleach and quickly frozen in liquid nitrogen. Protein extracts were prepared from ∼10 μL of embryos, as described for gonads.

Electrophoresis was carried out on 5–20% gradient SDS polyacrylamide gel and Western blotting was performed using the ECL prime Western blotting detection reagent and following the manufacturer's instructions (GE Healthcare). Uncropped images are in Supplementary Fig. 6.

The antibodies were: mouse monoclonal anti-α-Tubulin (Sigma #T9026, 1:500), rabbit polyclonal anti-DHD (1:1,000), rabbit polyclonal anti-Trx-2^51^ (1:1,000; a gift from Katja Becker), HRP-conjugated goat anti-mouse (Biorad #170-5047; 1:50,000) and peroxidase-conjugated goat anti-rabbit (Thermoscientific #32460; 1:20,000).

### RNA probe synthesis

A 436 bp *dhd* sequence was amplified using primers containing the T3 or T7 promoter sequences (*T3-dhd-f*: 5′-AATTAACCCTCACTAAAGGGCGACGACAAGCTAATCGTGC-3′, *T7-dhd-r*: 5′-TAATACGACTCACTATAGGGTACCCACATCTCGCGCTTAC-3′). A DIG labelled RNA antisense probe was directly transcribed from the purified PCR amplicon with T7 RNA polymerase and a labelling mix (Roche).

### RNA *in situ* hybridization

Ten pairs of ovaries from 1 to 5 day-old virgin females were dissected in 1X PBS; 0.2% Tween 20 (PBST), fixed for 30 min in a 1:1 mixture of 3.7% formaldehyde-PBST:heptane. After PBST washings, the tissues were post-fixed with 3.7% formaldehyde-PBST for 10 min, then washed in PBST, and sequentially incubated in 1:1 PBST/hybridization buffer (HB) (50% formamide, 5X SSC, 0.1% Tween 20, pH 6.5) for 10 min, in 100% HB for 10 min, and finally in HB containing 100 μg ml^−1^ tRNA and 50 μg ml^−1^ heparin, for 1 h at 65 °C. Then, the probe was added before overnight incubation at 65 °C. The ovaries were then washed twice with HB at 65 °C, and once with1:1 PBST:HB at 65 °C. DIG-labelled RNA probes detection was performed using anti-DIG AP antibody (Roche #11093274910; 1:100) and NBT/BCIP colorimetric detection was carried out according to manufacturer instructions (Roche). Ovaries were mounted in mounting medium (Dako) and were imaged using a Z1 microscope (Carl Zeiss).

### Data Availability

All relevant data are available from the authors on request.

## Additional information

**How to cite this article**: Tirmarche, S. *et al*. Unlocking sperm chromatin at fertilization requires a dedicated egg thioredoxin in *Drosophila. Nat. Commun.* **7**, 13539 doi: 10.1038/ncomms13539 (2016).

**Publisher's note**: Springer Nature remains neutral with regard to jurisdictional claims in published maps and institutional affiliations.

## Supplementary Material

Supplementary Information Supplementary Figures 1 - 9

Peer Review

## Figures and Tables

**Table 1:** Embryo hatching rates.

**Genotype of females**	**Genotype of males**	**No. of eggs**	**Hatch. rate (%)**
*w*^*1118*^	*w*^*1118*^*/Y*	264	90.9
w^*^, Df(1)dhd^*J5*^/ w^*^, Df(1)dhd^*J5*^	*w*^*1118*^*/Y*	577	0
w^*^, Df(1)dhd^*J5*^*/ Df(1)JC70*	*w*^*1118*^*/Y*	489	0
w^*^, Df(1)dhd^*J5*^/ w^*^, Df(1)dhd^*J5* ^*; CyO, P[dhd*^*XhoI/XhoI*^*, w*^*+*^*]/+*	*w*^*1118*^*/Y*	136	80.1
w^*^, Df(1)dhd^*J5*^/ w^*^, Df(1)dhd^*J5*^	*w*^*1118*^*/Y ; ΔMst35B/ΔMst35B*	328	0
w^*^, Df(1)dhd^*J5/*^ w^*^, Df(1)dhd^*J5*^ *; P[dhd*^*WT*^*,w*^*+*^*]/P[dhd*^*WT*^*,w*^*+*^*]*	*w*^*1118*^*/Y*	251	80.5
w^*^, Df(1)dhd^*J5*^/ w^*^, Df(1)dhd^*J5*^ *; P[dhd*^*C34S*^*,w*^*+*^*]/P[dhd*^*C34S*^*,w*^*+*^*]*	*w*^*1118*^*/Y*	955	0.2

The *Df(1)dhd*^*J5*^ deficiency and the *P[dhd*^*XhoI/XhoI*^*]* genomic rescue transgene are described in Fig. 1a. *Df(1)JC70* is a large deficiency uncovering the *dhd* locus. *ΔMst35B* mutation is described in ref. 39. *P[dhd*^*WT*^*]* and *P[dhd*^*C34S*^*]* transgenes are described in the text. w* indicates an unspecified mutant allele of the *white* gene.
