# Supplementary material for: Unlocking sperm chromatin at fertilization requires a dedicated egg thioredoxin in Drosophila
Source: Nat Commun. 2016 Nov 23;7:13539. doi: 10.1038/ncomms13539 (PMC5122968; doi:10.1038/ncomms13539)
Supplement: Supplementary Information — Supplementary Figures 1 - 9 [file ncomms13539-s1.pdf]

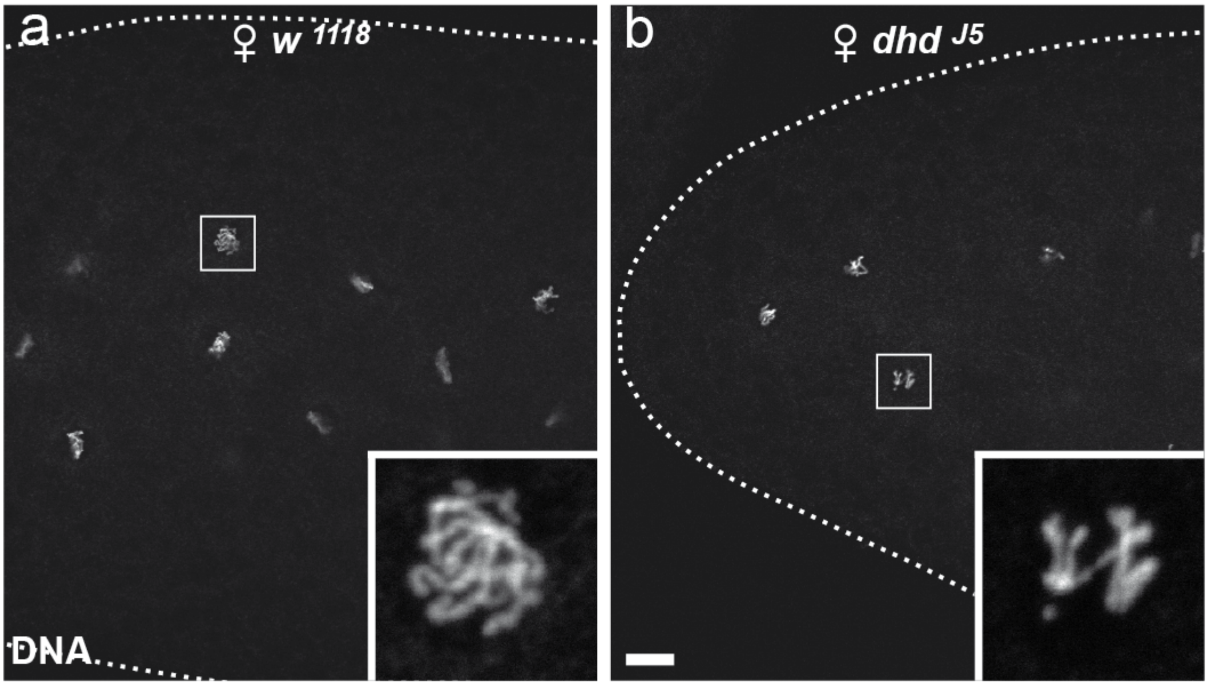

Supplementary Figure 1. *dhd* embryos are haploid. a) Confocal image of a wild-type syncytial embryo stained for DNA. Detail of a diploid nucleus (2n=8) in prometaphase is shown (inset). **b)** Confocal image of an early *dhd* embryo with haploid mitotic nuclei (n=4). Dotted lines indicate the outline of the embryos. Scale bar: 10 μm.

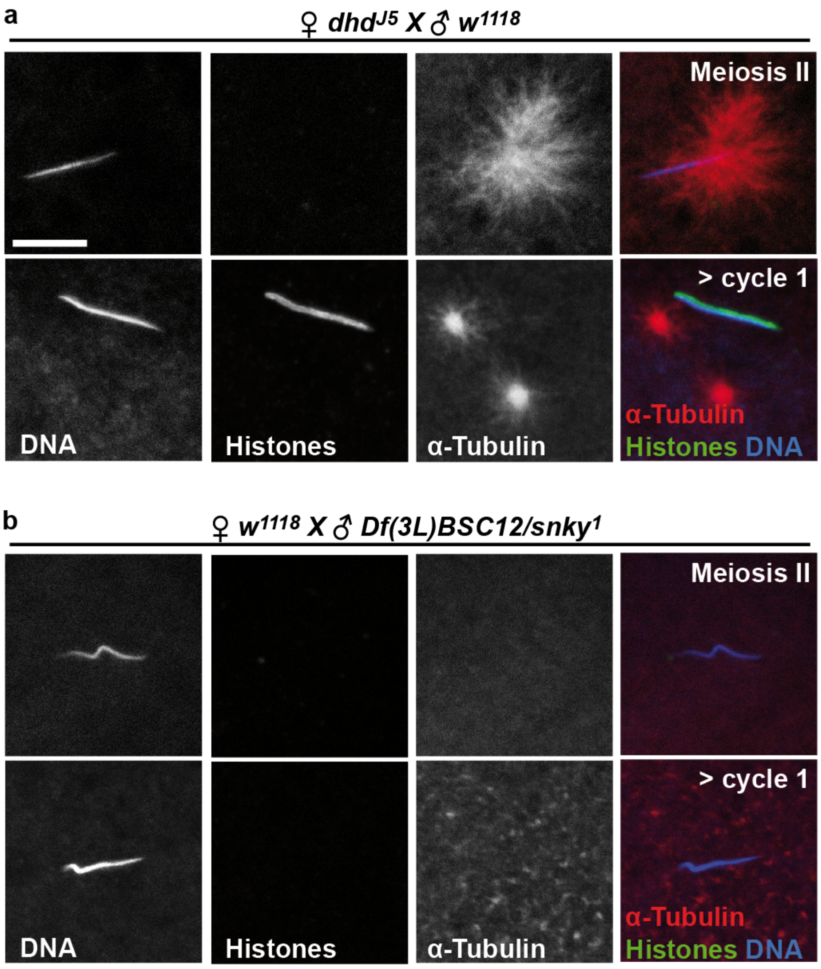

Supplementary Figure 2. Comparison of *snky* and *dhd* phenotypes. a) Egg/embryo from *dhd* mutant females crossed with wild-type males. Upper panels: In *dhd* eggs, the sperm aster is detected close to the needle-shaped nucleus. Lower panels: After the first nuclear cycle, histones become detectable in the sperm nucleus. **b)** Egg/embryo from wild-type females crossed with *snky* deficient males. Upper panels: The sperm aster is never observed in eggs fertilized by *snky* sperm. Lower panels: After cycle 1, histones are still not detected in the *snky* mutant sperm nucleus. Blue: DNA, green: histones, red: α-tubulin. Scale bar for a) and b): 5 μm.

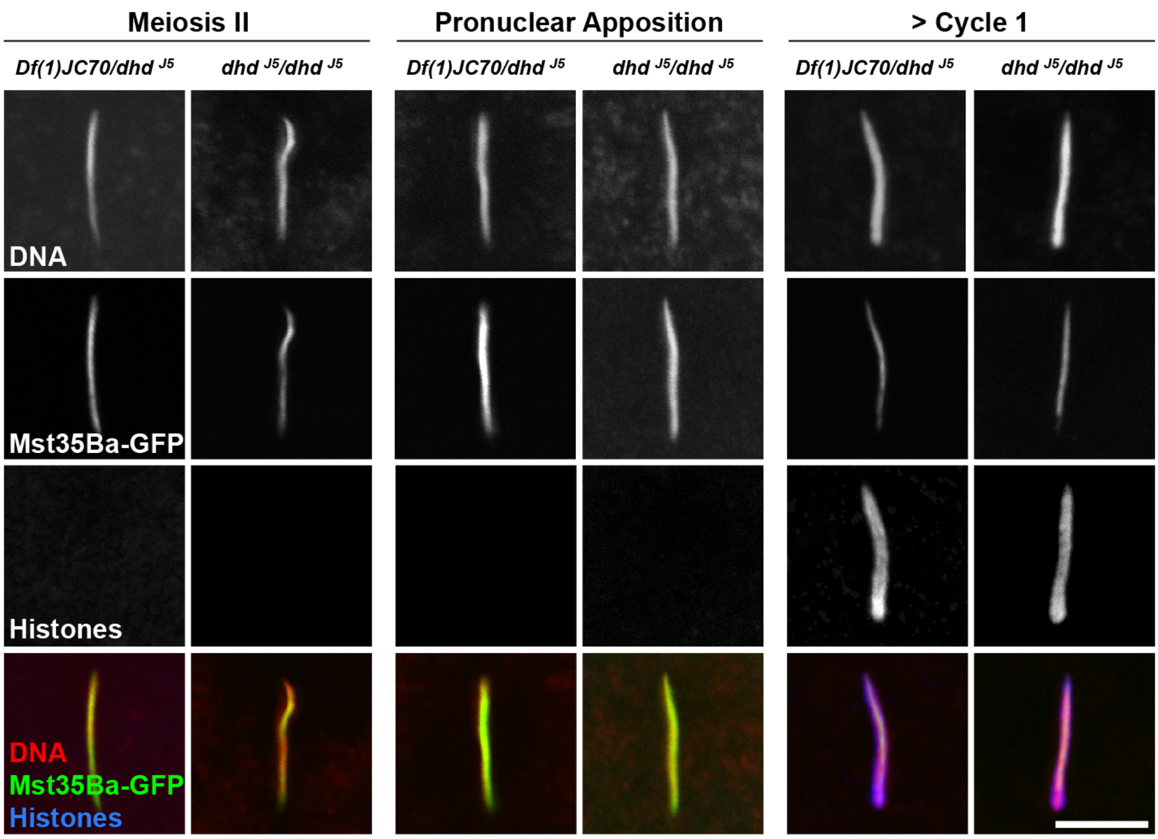

Supplementary Figure 3. Eggs from *Df(1)JC70/dhd^{J5}* and in *dhd^{J5}/dhd^{J5}* females display identical sperm chromatin remodeling defects. Confocal images of sperm nuclei from eggs/embryos laid by hemizygous *Df(1)JC70/dhd^{J5}* or homozygous *dhd^{J5}/dhd^{J5}* females mated with *Mst35Ba-GFP* transgenic males. Eggs or embryos at the indicated stages were stained for histones (blue) and DNA (red). GFP is in green. Scale bar: 5 µm.

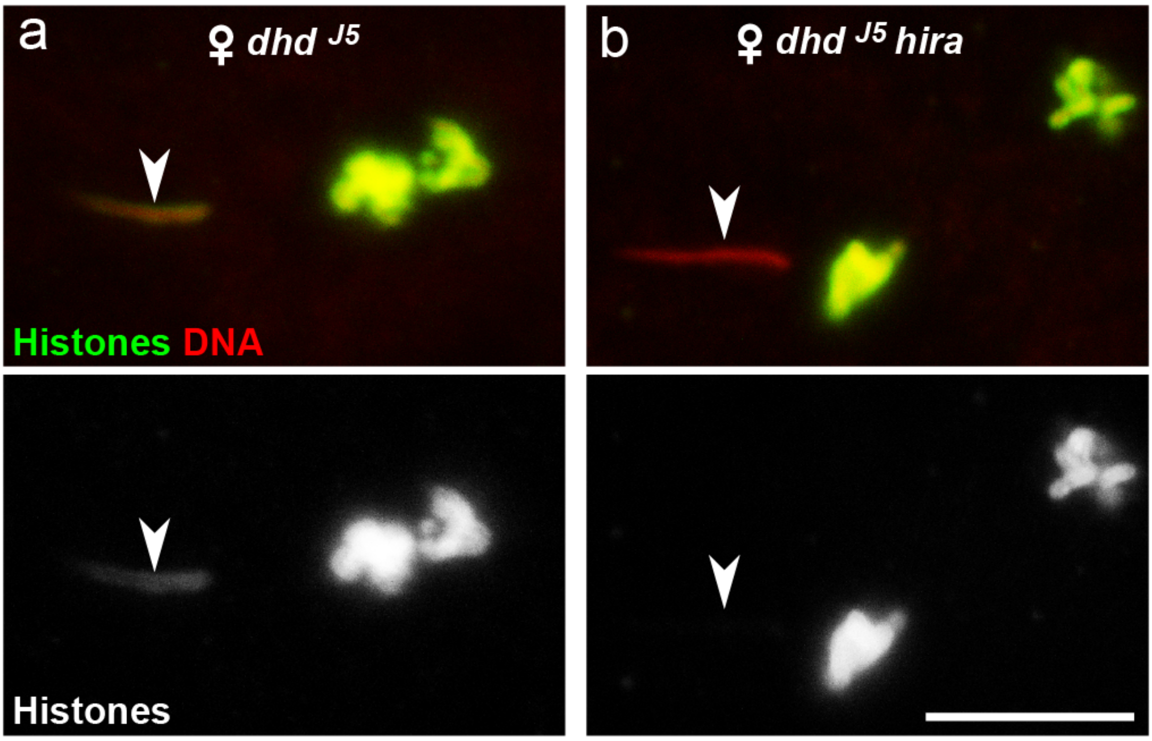

Supplementary Figure 4. In *dhd* embryos, histones are *de novo* deposited by the HIRA histone chaperone in the sperm nucleus. a) A cycle 2 *dhd* embryo showing limited histone deposition in the sperm nucleus (arrowhead). Note that the two haploid nuclei on the right appear close to one another in this projection of several images at different focal planes. (**b**) A cycle 2 embryo from a dhd^{J5} $hira^{ssm}$ double mutant female with undetectable level of histones in the sperm nucleus (arrowhead). Scale bar for a) and b): 10 μm.

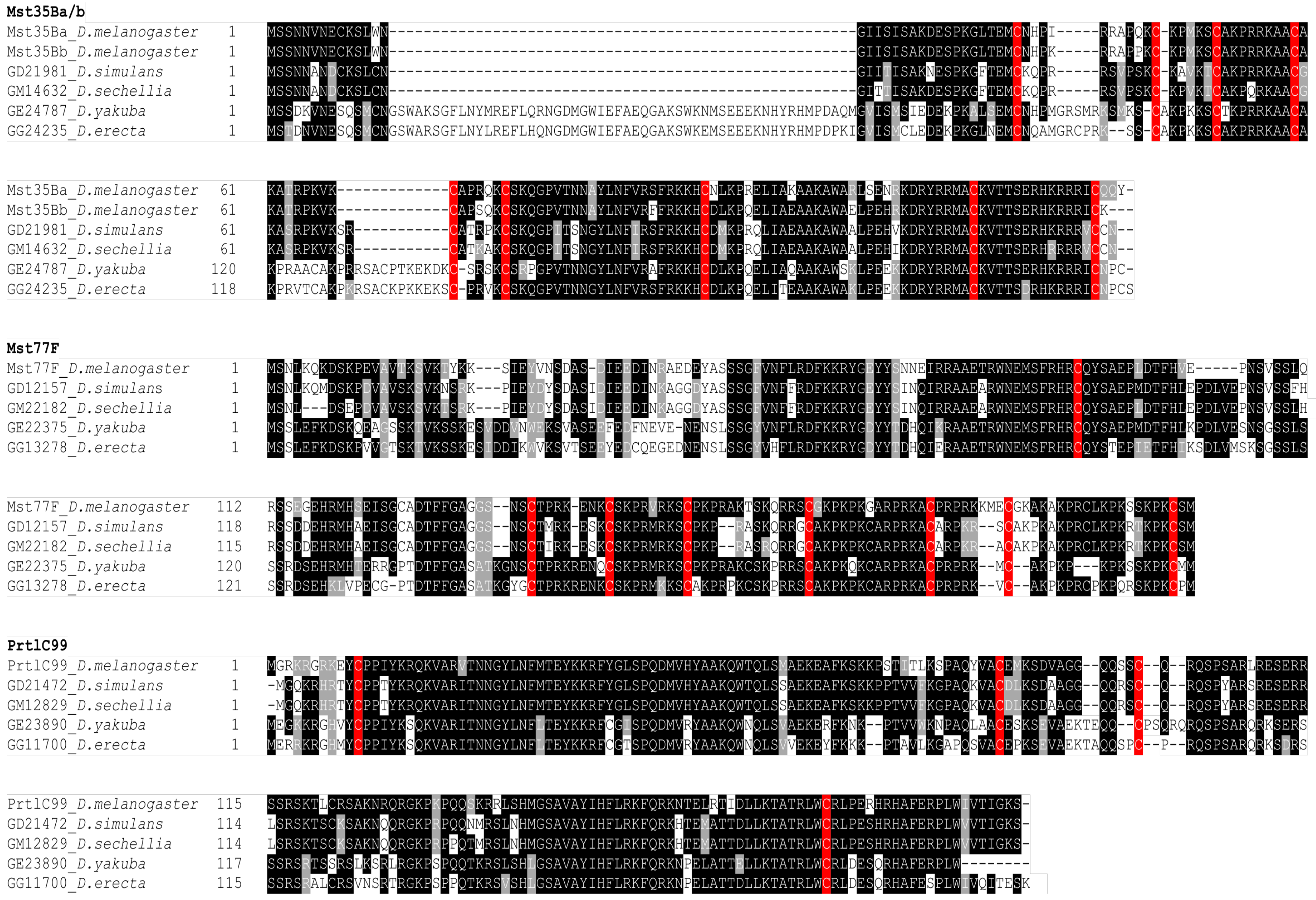
Mst35Ba/b
Mst35Ba_D.melanogaster 1 MSSNNVNECKSLWN--GIISISAKDESPKGLTEMCNHPI-----RRAPQKC-KPMKSCAKPRRKAACA
Mst35Bb_D.melanogaster 1 MSSNNVNECKSLWN--GIISISAKDESPKGLTEMCNHPK-----RRAPPKC-KPMKSCAKPRRKAACA
GD21981_D.simulans 1 MSSNNANDCKSLCN--GIITISAKNESPKGFTEMCKQPR-----RSVPSKC-KAVKTCAKPRRKAACG
GM14632_D.sechellia 1 MSSNNANDCKSLCN--GITTISAKDESPKGFTEMCKQPR-----RSVPSKC-KPVKTCAKPQRKAACG
GE24787_D.yakuba 1 MSSDKVNESQSMCNGSWAKSGFLNYMREFLQRNGDMGWIEFAEQGAKSWKNMSEEEKNHYRHMPDAQMGVISMSIEDEKPKALSEMCNHPMGRSMRKSVKS-CAKPKKSCTKPRRKAACA
GG24235_D.erecta 1 MSTDNVNESQSMCNGSWARSGFLNYLREFLHQNGDMGWIEFAEQGAKSWKEMSEEEKNHYRHMPDPKIGVISMCLEDEKPKGLNEMCNQAMGRCPRK--SS-CAKPKKSCAKPRRKAACA
Mst35Ba_D.melanogaster 61 KATRPKVK------------CAPRQKCSKQGPVTNNAYLNFVRSFRKKHCNLKPRELIAKAAKAWARLSENRKDRYRRMACKVTTSERHKRRRICQQY-
Mst35Bb_D.melanogaster 61 KATRPKVK------------CAPSQKCSKQGPVTNNAYLNFVRFFRKKHCDLKPQELIAEAAKAWAELPEHRKDRYRRMACKVTTSERHKRRRICK---
GD21981_D.simulans 61 KASRPKVKSR----------CATRPKCSKQGPITSNGYLNFIRSFRKKHCDMKPRQLIAEAAKAWAALPEHVKDRYRRMACKVTTSERHKRRRVCCN--
GM14632_D.sechellia 61 KASRPKVKSR----------CATKAKCSKQGPITSNGYLNFIRSFRKKHCDMKPRQLIAEAAKAWAALPEHIKDRYRRMACKVTTSERHRRRRVCCN--
GE24787_D.yakuba 120 KPRAACAKPRRSACPTKEKDKC-SRSKCSRPGPVTNNGYLNFVRAFRKKHCDLKPQELIAQAAKAWSKLPEEKKDRYRRMACKVTTSERHKRRRICNPC-
GG24235_D.erecta 118 KPRVTCAKPKRSACKPKKEKSC-PRVKCSKQGPVTNNGYLNFVRSFRKKHCDLKPQELITEAAKAWAKLPEEKKDRYRRMACKVTTSDRHKRRRICNPCS
Mst77F
Mst77F_D.melanogaster 1 MSNLKQKDSKPEVAVIKSVKTYKK---SIEYVNSDAS-DIEEDINRAEDEYASSSGFVNFLRDFKKRYGEYYSNNEIRRAAETRWNEMSFRHRCQYSAEPLDTFHVE-----PNSVSSLQ
GD12157_D.simulans 1 MSNLKQMDSKPDVAVSKSVKNSRK---PIEYDYSDASIDIEEDINKAGGDYASSSGFVNFFRDFKKRYGEYYSINQIRRAAEAARWNEMSFRHRCQYSAEPMDTFHLEPDLVEPNSVSSFH
GM22182_D.sechellia 1 MSNL---DSEPDVAVSKSVKTSRK---PIEYDYSDASIDIEEDINKAGGDYASSSGFVNFFRDFKKRYGEYYSINQIRRAAEAARWNEMSFRHRCQYSAEPLDTFHLEPDLVEPNSVSSLH
GE22375_D.yakuba 1 MSSLEFKDSKQEAGSSKTVKSSKESVDDVNWEKSVASEEFEDFNEVE-NENSLSSGYVNFLRDFKKRYGDYYTDHQIKRAAETRWNEMSFRHRCQYSAEPMDTFHLKPDLVESNSGSSLS
GG13278_D.erecta 1 MSSLEFKDSKPVVGTSKTVKSSKESIDDIKVVKSVTSEEYELCQEGEDNENSLSSGYVHFLRDFKKRYGDYYTDHQIERAAETRWNEMSFRHRCQYSTEPIETFHIKSDLVMSKSGSSLS
Mst77F_D.melanogaster 112 RSSEGEHRMHSEISGCADTFFGAGGS--NSCTPRK-ENKCSKPRVRKSCPKPRAKTSKQRRSCGKPKPKGARPRKACPRPRKKMECGKAKAKPRCLKPKSSKPKCSM
GD12157_D.simulans 118 RSSDDEHRMHAEISGCADTFFGAGGS--NSCTMRK-ESKCSKPRMRKSCPKP--RASKQRRGCAKPKPKCARPRKACARPKR--SCAKPKAKPRCLKPKRTKPKCSM
GM22182_D.sechellia 115 RSSDDEHRMHAEISGCADTFFGAGGS--NSCTIRK-ESKCSKPRMRKSCPKP--RASRQRRGCAKPKPKCARPRKACARPKR--ACAKPKAKPRCLKPKRIKPKCSM
GE22375_D.yakuba 120 SSRDSEHRMHTERRGPTDTFFGASATKGNSCTPRKRENQCSKPRMRKSCPKPRAKCSKPRRSCAKPKQKCARPRKACPRPRK--MC--AKPKP---KPKSSKPKCMM
GG13278_D.erecta 121 SSRDSEHKLVPECG-PTDTFFGASATKGYGCTPRKRENKCSKPRMKKSCAKPRPKCSKPRRSCAKPKPKCARPRKACPRPRK--VC--AKPKPRCPKPQRSKPKCPM
PrtlC99
PrtlC99_D.melanogaster 1 MGRKRGRKEYCPPIYKRQKVARVTNNGYLNFMTEYKKRFYGLSPQDMVHYAAKQWTQLSMAEKEAFKSKKPSTITLKSPAQYVACEMKSDVAGG--QQSSC--Q--RQSPSARLRESERR
GD21472_D.simulans 1 -MGQKRHRTYCPPTYKRQKVARITNNGYLNFMTEYKKRFYGLSPQDMVHYAAKQWTQLSSAEKEAFKSKKPPTVVEKGPAQKVACDLKSDAAGG--QQRSC--Q--RQSPYARSRESERR
GM12829_D.sechellia 1 -MGQKRHRTYCPPTYKRQKVARITNNGYLNFMTEYKKRFYGLSPQDMVHYAAKQWTQLSSAEKEAFKSKKPPTVVEKGPAQKVACDLKSDAAGG--QQRSC--Q--RQSPYARSRESERR
GE23890_D.yakuba 1 MEGKKRGHVYCPPIYKSQKVARITNNGYLNFITEYKKRFCGISPQDMVRYAAKQWNQLSVAEKERFKNK--PTVVWKNPAQLAACESKSFVAEKTEQQ--CPSQRQRQSPSARQRKSERS
GG11700_D.erecta 1 MERRKRGHMYCPPIYKSQKVARITNNGYLNFITEYKKRFCGTSPQDMVRYAAKQWNQLSVVEKEYFKKK--PTAVLKGAPQSVACEPKSFVAEKTAQQSPC--P--RQSPSARQRKSDRS
PrtlC99_D.melanogaster 115 SSRSKTLCRSAKNRQRGKPKPQQSKRRLSHMGSAVAYIHFLRKFQRKNTELRTIDLLKTATRLWCRLPERHRHAFERPLWIVTIGKS-
GD21472_D.simulans 114 LSRSKTSCKSAKNQQRGKPRPQQNMRSLNHMGSAVAYIHFLRKFQRKHTEMATTDLLKTATRLWCRLPESHRHAFERPLWVVTIGKS-
GM12829_D.sechellia 114 LSRSKTSCKSAKNQQRGKPRPPQTMRSLNHMGSAVAYIHFLRKFQRKHTEMATTDLLKTATRLWCRLPESHRHAFERPLWVVTIGKS-
GE23890_D.yakuba 117 SSRSRTSSRSLKSRLRGKPSPQQTKRSLSHLGSAVAYIHFLRKFQRKNPELATTELLKTATRLWCRLDESQRHAFERPLW--------
GG11700_D.erecta 115 SSRSRALCRSVNSRTRGKPSPPQTKRSVSHLGSAVAYIHFLRKFQRKNPELATTDLLKTATRLWCRLDESQRHAFESPLWIVQITESK

Supplementary Figure 5. Protein sequence alignments of SNBPs from five *Drosophila* species of the *melanogaster* subgroup. Identical (black) and similar (grey) amino acid residues are shown. Conserved cysteine residues are highlighted in red. Note that *D. simulans, D. sechellia, D. yakuba* and *D. erecta* have a single *Mst35B* gene.

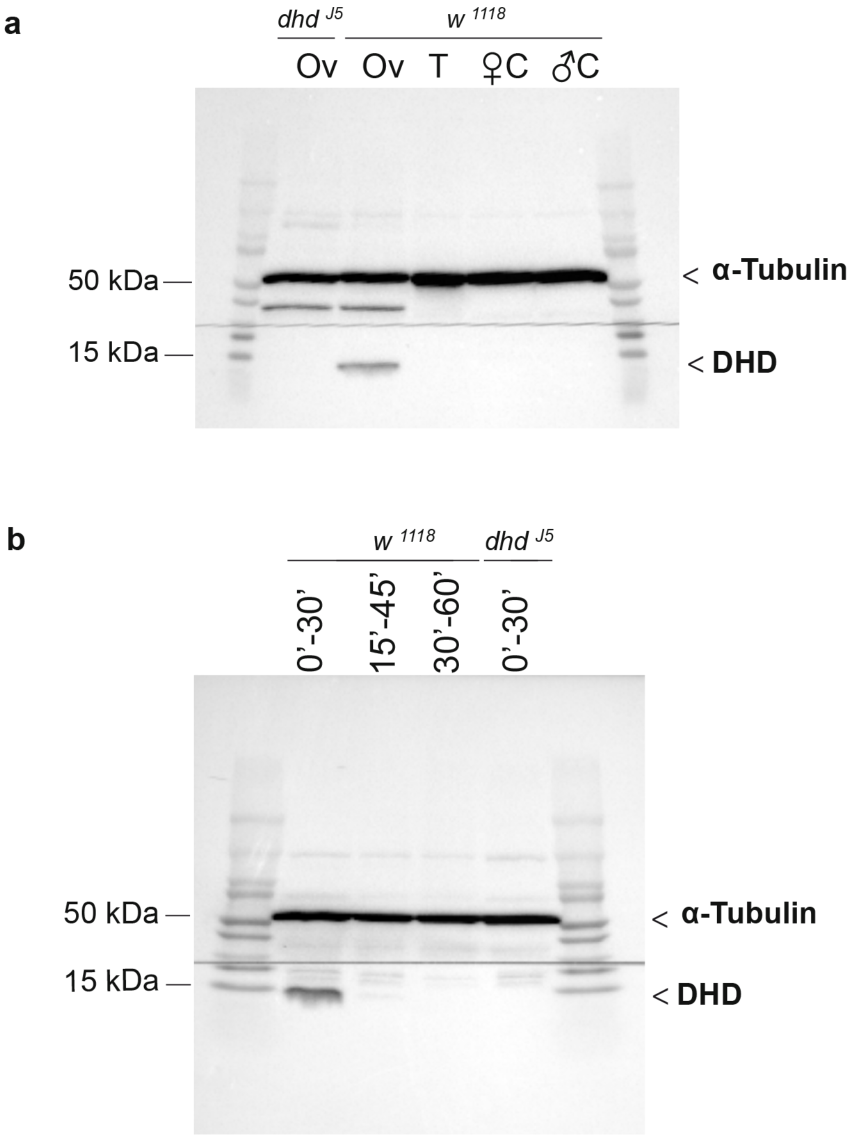

Supplementary Figure 6. Western blots of Figure 5

a) Full Western Blot of Fig. 5a.

b) Full Western Blot of Fig. 5b.

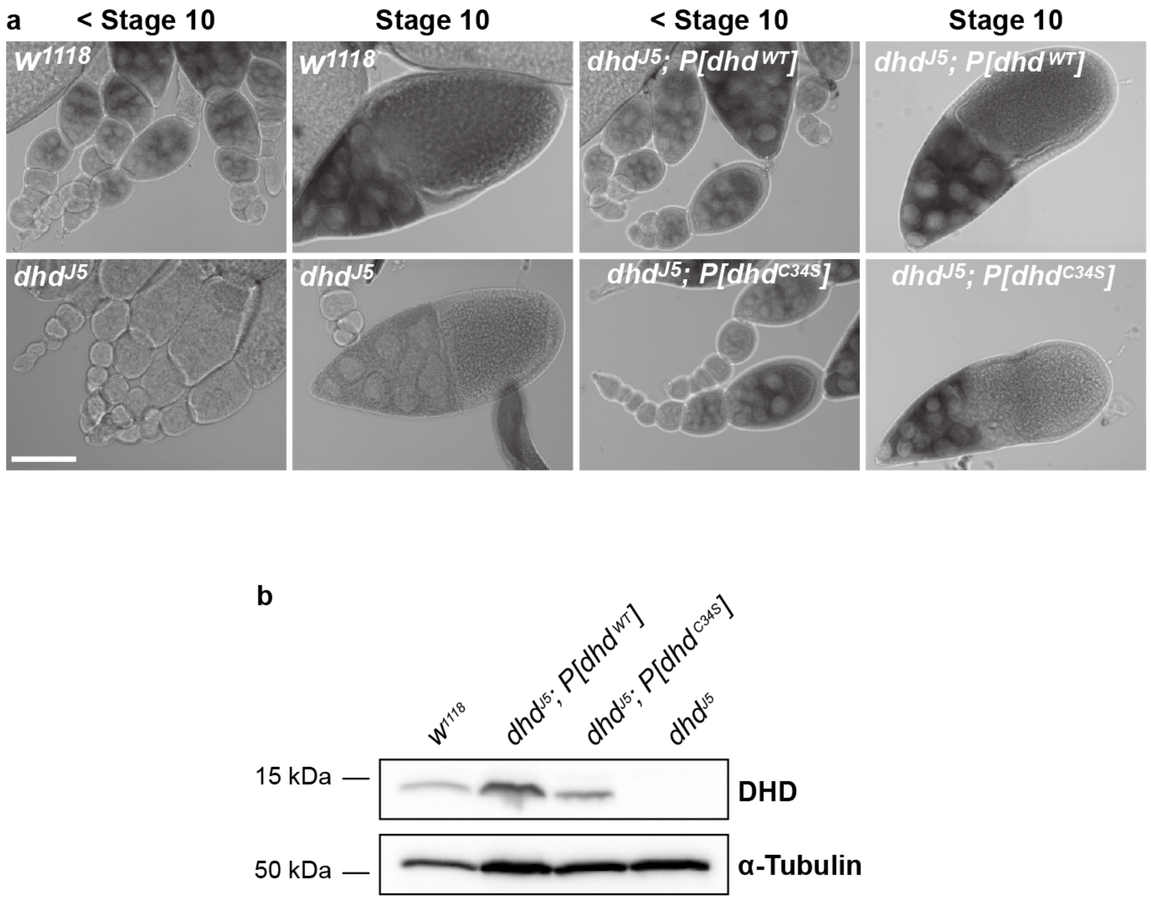

Supplementary Figure 7. Expression of the *P[dhd^{WT}]* and *P[dhd^{C34S}]* transgenes in ovaries. a) *dhd* mRNA *in situ* hybridization of ovarian chambers at the indicated stages. *dhd* mutant ovaries are used as a negative control for the *dhd* mRNA probe. **b)** Western blot analysis of DHD protein in *w^{1118}*, *dhd^{J5}*, *dhd^{J5}; P[dhd^{WT}]* and *dhd^{J5}; P[dhd^{C34S}]* ovarian extracts. Scale bar: 100 μm.

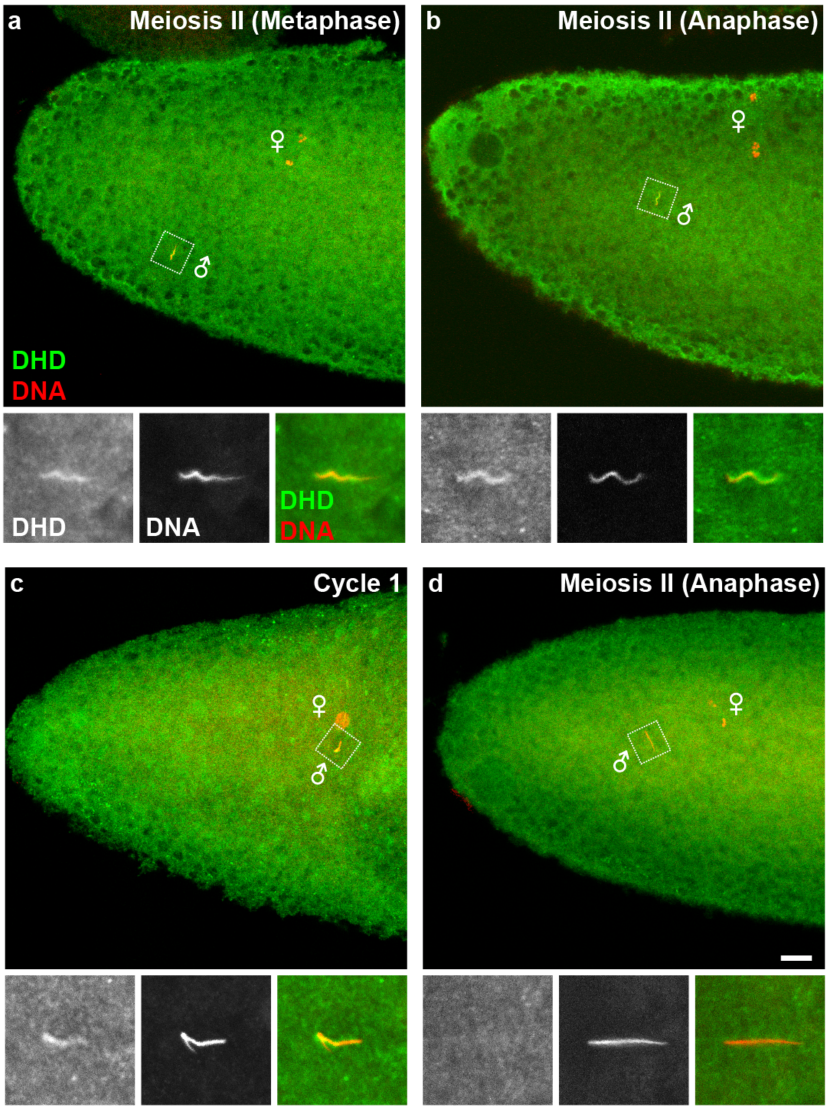

Supplementary Figure 8. DHD^{C34S} is specifically trapped on the sperm nucleus. Confocal images of eggs/embryos from *dhd^{J5}; P[dhd^{C34S}]* females stained for DHD (green) and DNA (red). Examples of eggs with sperm nuclei (insets) positive (**a-c,** 36%, n=36) or negative (**d)** for DHD. Scale bar: 10 µm.

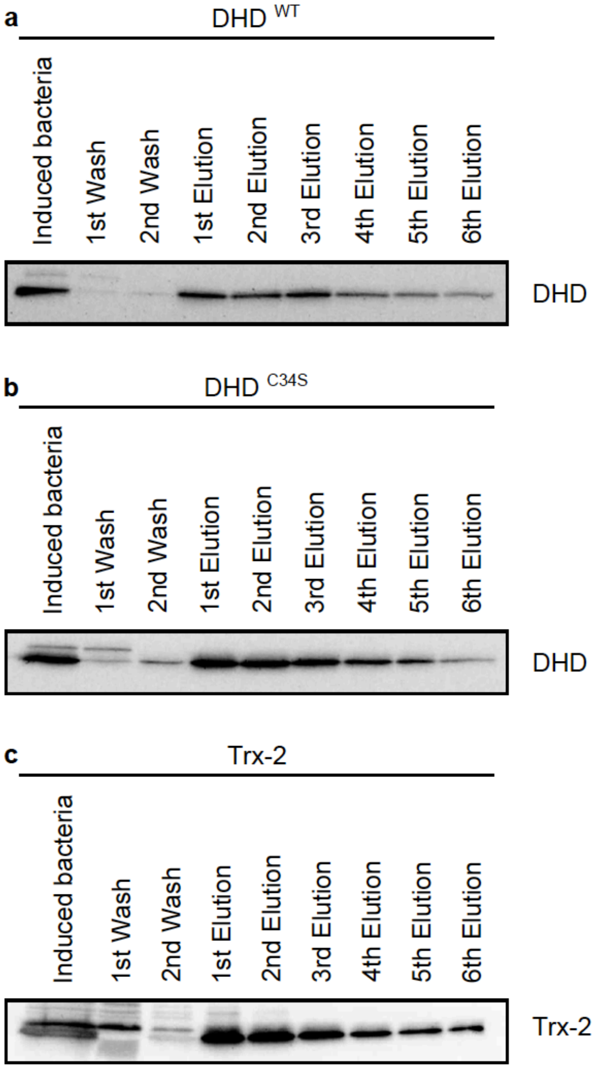

Supplementary Figure 9. Western-Blot analysis of purified recombinant thioredoxins. Each step of the His-tag purification procedure was analyzed by Western-Blotting using anti-DHD (**a, b**) or anti-Trx-2 (**c**) antibodies.
