# Supplementary material for: Unlocking sperm chromatin at fertilization requires a dedicated egg thioredoxin in Drosophila
Source: Nat Commun. 2016 Nov 23;7:13539. doi: 10.1038/ncomms13539 (PMC5122968; doi:10.1038/ncomms13539)
Supplement: Peer Review [file ncomms13539-s2.pdf]

PEER REVIEW FILE

Reviewers' comments:

Reviewer #1 (Remarks to the Author):

In this manuscript, Trimarche and colleagues report the identification and functional analyses of an enzyme which specifically disrupts disulfite bonds that crosslink sperm nuclear basic proteins (SNBPs).
An elegant set of experiments using appropriately designed models have generated clear-cut results supporting the idea that a Drosophila maternal thioredoxin, DHD, plays a critical role in male genome decondensation, SNBP removal and maternal histone assembly after fertilization. The use of transgenic flies expressing a mutant DHD designed to establish covalent enzymatic reaction intermediates with SNBPs clearly supports the action of this enzyme directly on SNBP. However, without a direct in vitro test, it would be impossible to definitely rule out an indirect effect. Therefore, the value of this manuscript would certainly increase if the authors could present data showing that DHD could mediate a DTT-type of sperm genome decompaction in vitro. This would of course require the successful production of a functional DHD for the assay. Although it would be worth that the authors try this approach, lack of success in this experiment should not hinder the publication of this fine piece of work.

Reviewer #2 (Remarks to the Author):

Tirmarche et al. show that the thioredoxin product of the Drosophila melanogaster deadhead (dhd) gene is necessary to decondense sperm chromatin in zygotes, and for the consequent participation of sperm chromosomes in the embryo cells. The authors show that in embryos from dhd mutant mothers, called dhd embryos, the sperm nucleus fails to decondense quickly. Most dhd embryos do not develop far. The 5% that do develop show defects consistent with haploidy. This phenotype led to the authors' insight that dhd affected the sperm's ability to participate in embryo development. This was a novel and important insight. I agree with the authors' conclusion in line 81 that they have shown that dhd is required for rapid removal of SNBPs. Using a GFP-MSt35Ba fusion, the authors show that a reducing agent could hasten removal of this SNBP from sperm in vitro. Taken together with the in vivo data this leads them to suggest that thioredoxin acts as a reducing agent to decondense sperm chromatin, letting it participate in the embryo. Dhd also appears necessary for sperm centriole function.

A lot of interesting information is packed into this short paper. The experimental design and data are excellent. However, as much as I liked the paper I found items that were not convincing (1-4) or not clear (5-7). If all are addressed satisfactorily in a revision, this article will be an excellent publication in Nature Communications.

1. In several instances of multiple phenotypes, the authors only discuss the small subset that supports their hypothesis. They need to explain why or, more importantly, how the other phenotypes do not rule out their hypothesis. Examples are:
Of the eggs that were fertilized, 5% of the embryos are haploid. The other 95% "failed to develop". What does this mean? Do they contain sperm nuclei that have not decondensed? Simply put, does the phenotype support the authors' hypothesis?
In 30% of dhd;P[dhdC34S] eggs the authors saw the mutant protein in a needle-shaped sperm nucleus. Why only 30%? Were the other 70% condensed but without obvious DHDC34S? If so, how to explain this? Or were they decondensed and without DHDC34S? Does this match the percentage of needle and decondensed in wild type?

2. That there is some histone deposition in paternal nuclei of dhd embryos suggests that DHD is not the only maternal component that can cause decondensation and removal of SNBPs. In that context I think the authors need to be very cautious in concluding that glutathione is not playing a role (end of abstract, end of p. 6).

3. An alternative hypothesis could explain the observations that the paternal nucleus cannot exchange its DNA binding proteins and decondense and that a good centrosome cannot organize from the male centriole: dhd could be needed for complete breakdown of the sperm membrane. This hypothesis needs to be ruled out to support the authors' main hypothesis.

4. The data shown in Fig. 4 are not in question, but I am not fully convinced that the fact that DHDC34S is seen in the condensed nuclei proves that "DHD directly reduces target disulfides on sperm chromatin". The observation is consistent with this conclusion but it relies on assumptions about the effects of the mutation, and is correlational.

5. The authors report, in contrast to a prior study, that dhd embryos complete meiosis. Their extended Figure 3 supports their conclusion. Why the difference from the prior study? And how frequent was this normal meiosis, was it only in a few embryos? If so that could reconcile the difference between the studies.

6. In Figure 3a, I did not understand the interpretation that the increased PI fluorescence indicated nuclear decondensation. The amount of DNA in the nuclei does not change. Are they measuring the area in which they see PI fluorescence? That would make sense, but it is not what the graph indicates.

7. In Figure 3c, I did not understand why the BRB fluorescence drops, after it increased.

Minor
8. It would have been nice to show the lack of DHDC34S in the decondensed nuclei.

9. Some figures need scale bars.

10. bp was inverted on line 317.

Reviewer #3 (Remarks to the Author):

Tirmarche et al. (Loppin's group) address an important question concerning fertilization. In many organisms the paternal genome is tighly condensed by non-histone proteins, which often contain a high number of cysteine residues. Before zygote formation, these highly basic non-histone chromosomal proteins need to be removed before histones can be loaded to the paternal genome. Thirmarche et al. used the excellent genetics of Drosophila and combined transgenes with fluorescent tags to address how removal of sperm proteins might be achieved. The focus of this work is the dhd encoded thioredoxin as a candidate protein, which might be involved in releasing paternal cysteine-rich chromatin components. It was known that DHD a thioredoxin (disulfide reductase) is maternally deposited and required for early embryonic development, but the mechanism was not clear.

Now, Tirmarche et al. analyzed this dhd encoded thioredoxin as a candidate protein which might open the tightly condensed sperm chromatin by resolving cysteine bridges after sperm entry into the egg.
They investigated dhd mutant eggs and analysed the behavior of wild-type sperm chromatin in this mutant background. Key findings are a delay of histone detection on the paternal genome.

In summary,
this is an elegant approach to address intra- and/or intermolecular cysteine bridges as being important for sperm chromatin condensation. Importantly, the authors identified DHD, a thioredoxin which is reponsible for at least partially desolving cysteine bridges after fertilization and before paternal and maternal genomes form the nucleus of the zygote. The delay of histone loading and removal of Mst77F and Mst35B in dhd mutant background results in lack of „fusion" of paternal and maternal pronuclei.

The data is technically sound. The paper provides strong evidence for its conclusions. The results are novel and the manuscript is important to scientists in the specific field.

At several points the text can be improved, in particular concerning recent puplications (see below), maybe the manuscript was in part too „streamlined" due to word restrictions.

Suggested improvements

Titel:
The manuscript describes that the loading of histones is more rapid in wild-type than in mutants lacking DHD, a thioredoxin. Later than mitotic cycle 1 also the paternal genome is histone based!

The titel should be adapted so that the delay of histone loading is evident:
„Rapid unlocking of sperm chromatin is ....."

Figure2b can be interpreted in two ways, both possibilities should be mentioned in the corresponding text.

a) In dhh mutants histones have access to the paternal genome though delayed, lack of protamines allows more access for maternal histones (authors statement)
b) Some residual histones could be detected by Dorus et al. Nature Genetics 2006 (Table S2) by mass spec analysis. This should be kept in mind. In my view, residual histones in the sperm chromatin might be better accessible if part of the predicted disulfide bonds in SNBPs are dissolved.

Figure 2c and corresponding text:
a) Loss of both protamines leads to more sensitivety to X-rays (Mst35B Rathke et al., 2010), this might indeed argue for less densly packed chromatin in accordance with elongated chromatin area when protamines are deleted (Eren-Ghiani et al., 2015).
These data should be mentioned.

b) Depite loss of Mst35B (protamines), males are fertile (Rathke et al., 2010; Tirmarche et al., 2014) thus, the fertilization assays for this manuscript were possible. Here, double mutants for dhd and Mst35B show quicker loading of histones than dhd mutants. In addition, in these double mutants paternal chromosomes (visible as chromomes in contrast to dhd single mutants) are Mst77F free and histone-based after cycle 1. Thus, the authors show that Mst77F seems to be better replaced when Mst35B (protamines) are deleted. Recently, removal of MSt77F and histone deposition were reported by Doyen et al. (Cell Reports 2015) to be dependent on the chromatin remodeller ISWI. Might it be that removal of protamines by the Thioredoxin DHD makes it easier to remove Mst77F and load histones by ISWI?
This should be discussed!

The extended data Figure 4, Text lines 101 - 104 need more discussion in the text:
This Figure shows all cysteins in SNBPs, but ignores the predicted domain structure such as HMG box (potentially DNA binding) in protamines and Prtl99C and an aberrant one in Mst77F (Doyen et al., Cell Reports 2015), putative DNA binding regions such as a central (Prtl99C, Eren-Ghiani et al., Cell reports 2015) or C-terminal LCR (Mst77F; Kost et al., NAR 2015)). For Mst77F furthermore, a coiled coil domain was predicted. For Mst77F, in vitro assays revealed DNA binding (c-LCR) and homodimerization (Coiled-coil) properties. Importantly, DNA and chromatin can be compacted in vitro in dependence of these domains (Kost et al., 2015).
It might well be, that after the DNA binding and part of the compaction is due to these domains while the cysteine bridges work on top of this either to stabilize the protein structure or by intermolecular interactions between individual SNBPs associated with different DNA helices.

Minor recommendations:
Legend to Fig1D line 261
(the marker for female chromosomes are „green" but in the overlay with DNA the maternal chromosomes appear yellow):
..staining (green, yellow in die overlay with DNA))....

Figure 3 and Text line 105 -113.

The DDT effect on „opening" the chromatin for accessibilty of an antibody against Ptrl99C (Eren-Ghiani et al., 2015), supports the conclusion of Tirmarche et al., that cysteine brigdes might be important to compact the paternal genome. This supportive should be mentioned!!

Tirmarche et al. - Response to the referees' comments

We sincerely thank the referees for their positive appreciation of our work and for their helpful comments and suggestions.

Reviewer #1:

In this manuscript, Tirmarche and colleagues report the identification and functional analyses of an enzyme which specifically disrupts disulfide bonds that crosslink sperm nuclear basic proteins (SNBPs).
An elegant set of experiments using appropriately designed models have generated clear-cut results supporting the idea that a Drosophila maternal thioredoxin, DHD, plays a critical role in male genome decondensation, SNBP removal and maternal histone assembly after fertilization.
The use of transgenic flies expressing a mutant DHD designed to establish covalent enzymatic reaction intermediates with SNBPs clearly supports the action of this enzyme directly on SNBP.
However, without a direct in vitro test, it would be impossible to definitely rule out an indirect effect. Therefore, the value of this manuscript would certainly increase if the authors could present data showing that DHD could mediate a DTT-type of sperm genome decompaction in vitro. This would of course require the successful production of a functional DHD for the assay. Although it would be worth that the authors try this approach, lack of success in this experiment should not hinder the publication of this fine piece of work.

We have indeed followed this important suggestion and tested recombinant DHD protein in our in vitro sperm nuclear decondensation assay. We have also produced and tested the C34S mutant version of DHD as well as Trx-2, another Drosophila thioredoxin with ubiquitous expression. The results showed very clearly that only wild-type DHD induced sperm nuclear decondensation and SNBP removal, in a way similar to DTT. We have thus included these results (Figure 7 and Supplementary Figure 8) in the revised version.

Reviewer #2:

Tirmarche et al. show that the thioredoxin product of the Drosophila melanogaster deadhead (dhd) gene is necessary to decondense sperm chromatin in zygotes, and for the consequent participation of sperm chromosomes in the embryo cells. The authors show that in embryos from dhd mutant mothers, called dhd embryos, the sperm nucleus fails to decondense quickly. Most dhd embryos do not develop far. The 5% that do develop show defects consistent with haploidy. This phenotype led to the authors' insight that dhd affected the sperm's ability to participate in embryo development. This was a novel and important insight. I agree with the authors' conclusion in line 81 that they have shown that dhd is required for rapid removal of SNBPs. Using a GFP-MSt35Ba fusion, the authors show that a reducing agent could hasten removal of this SNBP from sperm in vitro. Taken together with the in vivo data this leads them to suggest that thioredoxin acts as a reducing agent to decondense sperm chromatin, letting it

participate in the embryo. Dhd also appears necessary for sperm centriole function.

A lot of interesting information is packed into this short paper. The experimental design and data are excellent. However, as much as I liked the paper I found items that were not convincing (1-4) or not clear (5-7). If all are addressed satisfactorily in a revision, this article will be an excellent publication in Nature Communications.

1. In several instances of multiple phenotypes, the authors only discuss the small subset that supports their hypothesis. They need to explain why or, more importantly, how the other phenotypes do not rule out their hypothesis. Examples are:
Of the eggs that were fertilized, 5% of the embryos are haploid. The other 95% "failed to develop". What does this mean? Do they contain sperm nuclei that have not decondensed? Simply put, does the phenotype support the authors' hypothesis?

In this much longer revised version, we have tried to describe more clearly the dhd embryonic lethal phenotype and have included quantifications that were missing in the original version.
First, the sperm decondensation defect is a 100% penetrant phenotype (observed in all fertilized eggs). This is clearly mentioned in the Results (l. 92): " In striking contrast, the sperm nucleus systematically retained its original needle-shape in fertilized dhd eggs, showing no sign of decondensation (100%, n=66; Fig. 1c)."
Most dhd eggs (78%) do not develop because of the defective female pronuclear migration. In these cases, the sperm nucleus is typically found in the center of the egg while the four female nuclei (3 polar bodies + 1 female pronucleus) remain at the periphery. In the rest of eggs (22%), the female pronucleus migrates and haploid development is initiated but only 5% reach late embryogenesis.

In 30% of dhd;P[dhdC34S] eggs the authors saw the mutant protein in a needle-shaped sperm nucleus. Why only 30%? Were the other 70% condensed but without obvious DHDC34S? If so, how to explain this? Or were they decondensed and without DHDC34S? Does this match the percentage of needle and decondensed in wild type?

As we tried to better explain here, the P[dhdC34S] transgene has no effect on the dhd sperm nuclear phenotype and all dhd;P[dhdC34S] eggs contain a needle-shape sperm nucleus. Among these, 30% are stained with the anti-DHD antibody the others are not stained. The absence of detectable staining in the other eggs is a matter of speculation. We have proposed that incomplete sperm plasma membrane breakdown could prevent access of DHD^{C34S} in these cases (l.228). Note that needle-shaped nuclei are never observed in the rescue with the wild-type transgene.

2. That there is some histone deposition in paternal nuclei of dhd embryos suggests that DHD is not the only maternal component that can cause decondensation and removal of SNBPs. In that context I think the authors need to be very cautious in concluding that glutathione is not playing a role (end of abstract, end of p. 6).

We entirely agree with this remark and we think that it is actually likely that glutathione is

responsible for the slow decondensation and SNBP removal observed in mutant eggs. We have specifically discussed this point in the discussion of the revised manuscript (l. 303).

3. An alternative hypothesis could explain the observations that the paternal nucleus cannot exchange its DNA binding proteins and decondense and that a good centrosome cannot organize from the male centriole: dhd could be needed for complete breakdown of the sperm membrane. This hypothesis needs to be ruled out to support the authors' main hypothesis.

This is a very important point indeed and we have tried to address it experimentally.
First, we directly compared the dhd phenotype with the paternal effect sneaky phenotype, where sperm membrane breakdown fails (Fitch and Wakimoto, Dev Biol, 1998). Although superficially similar, the phenotypes are clearly distinct since a sperm aster is systematically observed in dhd eggs, but is never present in eggs fertilized by snky sperm (Supplementary Figure 2). Similarly, histones are only detected in the sperm nucleus in dhd eggs, not snky. So it is clear that sperm plasma membrane breakdown (SPMB) is at least initiated in dhd eggs.
However, we also observed that the acrosome (detected with Snky-GFP; Wilson et al., Development, 2006) remains attached to the sperm nucleus in dhd eggs, instead of detaching as in wild-type eggs (Figure 2b of the revised manuscript). Unfortunately, our attempt to directly detect the membrane (using the CD2 marker) failed (we could not detect it in our control CD2; snky fertilized eggs).
We have thus discussed the possibility that the membrane is not completely removed in a fraction of dhd eggs. However, 1) the female pronuclear migration defect is not fully penetrant, 2) the DHD^{C34S} protein is specifically trapped on the sperm nucleus (and not the tail or acrosomal regions) and 3) the partial rescue of dhd with ΔMst35B sperm is difficult to explain in the membrane hypothesis. Finally, we have now included additional data demonstrating that DHD is sufficient to induce sperm nuclear decondensation in vitro.
In conclusion, we do not exclude the possibility that SPMB is incomplete in mutant eggs, and we have make this statement in the revised manuscript. This could explain for instance the defective separation of centrioles or acrosome from the nucleus, and the fact that DHD^{C34S} does not stain all nuclei. However, we think that our data altogether support the role of DHD in the cleavage of sperm chromatin disulfide bonds.

4. The data shown in Fig. 4 are not in question, but I am not fully convinced that the fact that DHDC34S is seen in the condensed nuclei proves that "DHD directly reduces target disulfides on sperm chromatin". The observation is consistent with this conclusion but it relies on assumptions about the effects of the mutation, and is correlational.

We agree that this result does not constitute a definitive evidence and we have changed the text accordingly (l. 230): " These results however strongly suggest that DHD directly reduces target disulfides present on sperm chromatin."

5. The authors report, in contrast to a prior study, that dhd embryos complete meiosis. Their extended Figure 3 supports their conclusion. Why the difference from the prior study? And how frequent was this normal meiosis, was it only in a few embryos? If so that could reconcile the difference between the studies.

We have provided additional data showing that meiosis occurred normally in 100% of dhd eggs (n=17). In the original study (Salz et al., 1994), the authors concluded that dhd eggs failed to complete meiosis based on the following indirect observations (no meiotic figures are shown in their paper):
- most dhd eggs fail to initiate embryonic divisions: We agree.
- most dhd eggs are fertilized: We agree.
- the female pronucleus is rarely observed: We agree (it is formed but remains at the periphery).
- Polar bodies are rarely observed, have too many chromosomes, or look abnormal. We agree: They are indeed frequently tetraploids ("too many chromosomes"). Also, from our long experience with egg phenotypes, we know that when eggs fail to develop, they rapidly show signs of nuclear degradation, especially if standard egg collection procedures are used (instead of very rapid collections). We think that Salz et al. used standard embryo procedures (the method they used is not provided), which would explains the fact that they could not directly observe meiosis (which is rapidly completed) and that many eggs show signs of degradation (explaining the abnormal appearance of the polar body in many eggs). In conclusion, the difference between these studies lies in the interpretation of the phenotype, not on the observations (with the exception of the sperm nucleus phenotype they missed). We have previous experience with female meiotic defects in Drosophila (see for instance Orsi et al., J. Cell Sci, 2010). Clearly, meiosis in dhd eggs occurs normally but female pronuclear migration frequently fails.

6. In Figure 3a, I did not understand the interpretation that the increased PI fluorescence indicated nuclear decondensation. The amount of DNA in the nuclei does not change. Are they measuring the area in which they see PI fluorescence? That would make sense, but it is not what the graph indicates.

Propidium Iodide poorly stains ultracompacted sperm nuclei but the staining increases with nuclear decompaction. We have used this simple parameter to evaluate nuclear decompaction. We also erroneously referred to Fig 4a (Fig. 3a in the original version) instead of Fig. 4d for the images of nuclear decompaction. We apologize for this mistake.

7. In Figure 3c, I did not understand why the BRB fluorescence drops, after it increased.

mBrB recognizes free thiol groups and becomes fluorescent. In our interpretation, the initial increase of mBrB fluorescence on DTT-treated sperm nuclei reflects the formation of free thiol groups after the cleavage of disulfide bonds connecting SNBPs. After a longer incubation in DTT, the cleavage of additional disulfide bonds facilitates the eviction of mBrB-bound SNBPs from sperm nuclei during the washing steps (Mst35Ba-GFP fluorescence indeed decreases), resulting in the observed decrease of mBrB fluorescence.

Minor
8. It would have been nice to show the lack of DHDC34S in the decondensed nuclei.

As explained above, DHD^{C34S} does not induce any sperm nuclear decondensation. We apologize for the confusion. We hope that this is now clear in the revised manuscript.

9. Some figures need scale bars.

These have been added.

10. bp was inverted on line 317.

Thank you.

Reviewer #3

Tirmarche et al. (Loppin's group) address an important question concerning fertilization. In many organisms the paternal genome is tightly condensed by non-histone proteins, which often contain a high number of cysteine residues. Before zygote formation, these highly basic non-histone chromosomal proteins need to be removed before histones can be loaded to the paternal genome. Tirmarche et al. used the excellent genetics of Drosophila and combined transgenes with fluorescent tags to address how removal of sperm proteins might be achieved. The focus of this work is the dhd encoded thioredoxin as a candidate protein, which might be involved in releasing paternal cysteine-rich chromatin components. It was known that DHD a thioredoxin (disulfide reductase) is maternally deposited and required for early embryonic development, but the mechanism was not clear.

Now, Tirmarche et al. analyzed this dhd encoded thioredoxin as a candidate protein which might open the tightly condensed sperm chromatin by resolving cysteine bridges after sperm entry into the egg.
They investigated dhd mutant eggs and analysed the behavior of wild-type sperm chromatin in this mutant background. Key findings are a delay of histone detection on the paternal genome.

In summary,
This is an elegant approach to address intra- and/or intermolecular cysteine bridges as being important for sperm chromatin condensation. Importantly, the authors identified DHD, a thioredoxin which is reponsible for at least partially desolving cysteine bridges after fertilization and before paternal and maternal genomes form the nucleus of the zygote. The delay of histone loading and removal of Mst77F and Mst35B in dhd mutant background results in lack of „fusion" of paternal and maternal pronuclei.

The data is technically sound. The paper provides strong evidence for its conclusions. The results are novel and the manuscript is important to scientists in the specific field.

At several points the text can be improved, in particular concerning recent puplications (see below), maybe the manuscript was in part too „streamlined" due to word restrictions.

Suggested improvements

Title:
The manuscript describes that the loading of histones is more rapid in wild-type than in

mutants lacking DHD, a thioredoxin. Later than mitotic cycle 1 also the paternal genome is histone based!

The title should be adapted so that the delay of histone loading is evident:
„Rapid unlocking of sperm chromatin is ....."

That is correct and we have discussed this point in the Discussion. However, a normal looking male pronucleus is never observed in mutant eggs. We thus feel that the original title, which underlines the critical implication of a thioredoxin in this process, rather than the dynamic aspect of the phenotype, is better suited. We however leave this at the appreciation of the editor.

Figure2b can be interpreted in two ways, both possibilities should be mentioned in the corresponding text.

a) In dhd mutants histones have access to the paternal genome though delayed, lack of protamines allows more access for maternal histones (authors statement)
b) Some residual histones could be detected by Dorus et al. Nature Genetics 2006 (Table S2) by mass spec analysis. This should be kept in mind. In my view, residual histones in the sperm chromatin might be better accessible if part of the predicted disulfide bonds in SNBPs are dissolved.

That is correct. We have thus generated dhd^{J5} $Hira^{ssm}$ double mutant females to prevent maternal deposition of histones on the sperm nucleus by the Hira chaperone (Supplementary Figure 4). Indeed, in eggs of double mutant females, histones are no longer detected in the sperm nucleus, thus demonstrating that all detected histones in our experiments are maternally deposited. We cannot exclude the presence of residual histones in sperm, but they remain undetectable in our hands (with the exception of Cid).

Figure 2c and corresponding text:
a) Loss of both protamines leads to more sensitivety to X-rays (Mst35B Rathke et al., 2010), this might indeed argue for less densly packed chromatin in accordance with elongated chromatin area when protamines are deleted (Eren-Ghiani et al., 2015). These data should be mentioned.

We agree. One of these papers had not been cited in the original submission because of constraints of the number of citations. We have now cited both studies (l. 169) so the reader can easily access this information. However, we do not directly mention the data supporting the less densely packed chromatin of this mutant to keep the text focused on dhd.

b) Despite loss of Mst35B (protamines), males are fertile (Rathke et al., 2010; Tirmarche et al., 2014) thus, the fertilization assays for this manuscript were possible. Here, double mutants for dhd and Mst35B show quicker loading of histones than dhd mutants. In addition, in these double mutants paternal chromosomes (visible as chromosomes in contrast to dhd single mutants) are Mst77F free and histone-based after cycle 1. Thus, the authors show that Mst77F seems to be better replaced when Mst35B (protamines) are deleted. Recently, removal of Mst77F and histone deposition were reported by Doyen et al. (Cell Reports 2015) to be dependent on the chromatin remodeller ISWI.

Might it be that removal of protamines by the Thioredoxin DHD makes it easier to remove Mst77F and load histones by ISWI?

This should be discussed!

We have no evidence supporting a specific role of DHD on Mst35Ba/b relative to Mst77F. However, we entirely agree that DHD is only expected to cleave disulfide bonds (including on Mst77F) while the eviction of SNBPs per se requires a distinct activity, such as ISWI, for instance. We have discussed this point in the Discussion (l. 284):

The extended data Figure 4, Text lines 101 - 104 need more discussion in the text:
This Figure shows all cysteines in SNBPs, but ignores the predicted domain structure such as HMG box (potentially DNA binding) in protamines and Prtl99C and an aberrant one in Mst77F (Doyen et al., Cell Reports 2015), putative DNA binding regions such as a central (Prtl99C, Eren-Ghiani et al., Cell reports 2015) or C-terminal LCR (Mst77F; Kost et al., NAR 2015)). For Mst77F furthermore, a coiled coil domain was predicted. For Mst77F, in vitro assays revealed DNA binding (c-LCR) and homodimerization (Coiled-coil) properties. Importantly, DNA and chromatin can be compacted in vitro in dependence of these domains (Kost et al., 2015).
It might well be, that after the DNA binding and part of the compaction is due to these domains while the cysteine bridges work on top of this either to stabilize the protein structure or by intermolecular interactions between individual SNBPs associated with different DNA helices.

The point of this Supplementary Figure (now Suppl. Fig. 5) was to show the presence of conserved cysteines in all fly SNBPs. Without exactly knowing were and how putative disulfide bonds are formed, we do not wish to discuss further the individual properties of these proteins as we feel that this is slightly out of the scope of our paper.
However, we now better describe the characteristics of fly SNBPs in the introduction and proposed that disulfide bonds could stabilize Mst77F multimerization, as suggested.

Minor recommendations:
Legend to Fig1D line 261
(the marker for female chromosomes are „green" but in the overlay with DNA the maternal chromosomes appear yellow):
..staining (green, yellow in die overlay with DNA))....

We have clarified the legend accordingly.

Figure 3 and Text line 105 -113.
The DDT effect on „opening" the chromatin for accessibility of an antibody against Ptrl99C (Eren-Ghiani et al., 2015), supports the conclusion of Tirmarche et al., that cysteine brigdes might be important to compact the paternal genome. This supportive should be mentioned!!

Thank you for having pointed this interesting fact that indeed nicely supports our own results. We have mentioned this point in the Discussion (l. 257).

Reviewers' Comments:

Reviewer #1 (Remarks to the Author):

None

Reviewer #2 (Remarks to the Author):

The added data, corrections, and clarifications have addressed my concerns. The new data are convincing, and so are the overall arguments now. The study is novel, carefully done, and important. The data quality is excellent. I fully support publication.

Reviewer #3 (Remarks to the Author):

Review to revison

The authors answered my questions in their rebuttal letter, added extended Figure 4, several references of importance to the data set are mentioned now.
I suggested a modification of the title, but also agree with the current and previous title following the choice of focus of the authors.

In summary this is a very nice piece of work with general relevance,high quality of data and clarity in presentation. I strongly recommend publication without additional changes.
